# FDA-approved cannabidiol [Epidiolex^®^] alleviates Gulf War Illness-linked cognitive and mood dysfunction, hyperalgesia, neuroinflammatory signaling, and declined neurogenesis

**DOI:** 10.1186/s40779-024-00563-2

**Published:** 2024-08-22

**Authors:** Maheedhar Kodali, Leelavathi N. Madhu, Venkata Sai Vashishta Kolla, Sahithi Attaluri, Charles Huard, Yogish Somayaji, Bing Shuai, Chase Jordan, Xiaolan Rao, Sanath Shetty, Ashok K. Shetty

**Affiliations:** Institute for Regenerative Medicine, Department of Cell Biology and Genetics, Texas A&M University Health Science Center School of Medicine, College Station, TX 77843 USA

**Keywords:** Gulf War Illness (GWI), Anhedonia, Activated microglia, Cannabidiol (CBD), Chronic neuroinflammation, Cognition, Hippocampal neurogenesis, Inflammasomes, Janus kinase/signal transducers and activators of the transcription (JAK/STAT) signaling, Memory dysfunction, NOD-, LRR- and pyrin domain-containing protein 3 (NLRP3) inflammasomes, Oxidative stress

## Abstract

**Background:**

Chronic Gulf War Illness (GWI) is characterized by cognitive and mood impairments, as well as persistent neuroinflammation and oxidative stress. This study aimed to investigate the efficacy of Epidiolex^®^, a Food and Drug Administration (FDA)-approved cannabidiol (CBD), in improving brain function in a rat model of chronic GWI.

**Methods:**

Six months after exposure to low doses of GWI-related chemicals [pyridostigmine bromide, *N,N*-diethyl-meta-toluamide (DEET), and permethrin (PER)] along with moderate stress, rats with chronic GWI were administered either vehicle (VEH) or CBD (20 mg/kg, oral) for 16 weeks. Neurobehavioral tests were conducted on 11 weeks after treatment initiation to evaluate the performance of rats in tasks related to associative recognition memory, object location memory, pattern separation, and sucrose preference. The effect of CBD on hyperalgesia was also examined. The brain tissues were processed for immunohistochemical and molecular studies following behavioral tests.

**Results:**

GWI rats treated with VEH exhibited impairments in all cognitive tasks and anhedonia, whereas CBD-treated GWI rats showed improvements in all cognitive tasks and no anhedonia. Additionally, CBD treatment alleviated hyperalgesia in GWI rats. Analysis of hippocampal tissues from VEH-treated rats revealed astrocyte hypertrophy and increased percentages of activated microglia presenting NOD-, LRR- and pyrin domain-containing protein 3 (NLRP3) complexes as well as elevated levels of proteins involved in NLRP3 inflammasome activation and Janus kinase/signal transducers and activators of the transcription (JAK/STAT) signaling. Furthermore, there were increased concentrations of proinflammatory and oxidative stress markers along with decreased neurogenesis. In contrast, the hippocampus from CBD-treated GWI rats displayed reduced levels of proteins mediating the activation of NLRP3 inflammasomes and JAK/STAT signaling, normalized concentrations of proinflammatory cytokines and oxidative stress markers, and improved neurogenesis. Notably, CBD treatment did not alter the concentration of endogenous cannabinoid anandamide in the hippocampus.

**Conclusions:**

The use of an FDA-approved CBD (Epidiolex^®^) has been shown to effectively alleviate cognitive and mood impairments as well as hyperalgesia associated with chronic GWI. Importantly, the improvements observed in rats with chronic GWI in this study were attributed to the ability of CBD to significantly suppress signaling pathways that perpetuate chronic neuroinflammation.

**Supplementary Information:**

The online version contains supplementary material available at 10.1186/s40779-024-00563-2.

## Background

Brain dysfunction is a prominent concern among the various health issues associated with Gulf War Illness (GWI). GWI affects over a third of the 700,000 veterans who served in the first Gulf War [1–6]. The brain-related problems encompass difficulties in forming new memories, recalling recently acquired memories, maintaining concentration, regulating mood, and achieving restful sleep [7, 8]. The most probable cause of GWI is varying levels of exposure to two or more chemicals during the war, including the nerve gas prophylactic drug pyridostigmine bromide (PB), insect repellent N,N-diethyl-m-toluamide (DEET), insecticide permethrin (PER), and pesticides [9, 10]. Other potential factors contributing to GWI include low-level exposure to sarin gas, combustion products, fuels from burning oil wells, mustard gas, vaccinations, and depleted uranium [2, 6, 11]. However, based on epidemiological and animal model studies, it is widely thought that GWI in a significant percentage of veterans is an outcome of exposure to PB, insecticides, and/or pesticides.

Due to the probable exposure of veterans to both GWI-related chemicals and war-induced stress, it is acknowledged that the neurological symptoms exhibited by a significant percentage of GW veterans are attributed to the synergistic interaction between a mixture of GWI-related chemicals and stress [1, 12, 13]. Consequently, investigations into the delayed effects of exposure to GWI-related chemicals with or without stress in animal models have garnered attention [14–21]. Studies conducted on mice have demonstrated that prolonged exposure to higher doses of PB and PER for 10 d can result in long-term memory impairments, astrocyte hypertrophy, synapse loss, and alterations in proteins related to lipid metabolism, molecular transport, and the endocrine and immune systems [22–27]. Meanwhile, studies performed on rats have indicated that exposure to low doses of PB, PER, and DEET, along with mild or moderate restraint stress for 4 weeks, can induce chronic oxidative stress and neuroinflammation, leading to persistent cognitive and mood impairments [14–18, 28–31]. Furthermore, a study utilizing positron emission tomography imaging using [^11^C]PBR28 on veterans diagnosed with GWI also suggested the presence of chronic inflammation in their brains [32].

Recently conducted studies in our laboratory using a rat model of chronic GWI have unveiled that exposure to low doses of PB, PER, and DEET for 4 weeks, accompanied by moderate stress (15 min of restraint stress), leads to enduring impairments in cognitive, memory, and mood [28, 30, 31, 33]. These impairments observed in GWI rats were notably linked to multiple neuropathological changes in the hippocampus. These alterations include: 1) an increase in astrocyte hypertrophy; 2) elevated percentages of activated microglia presenting NOD-, LRR- and pyrin domain-containing protein 3 (NLRP3) inflammasome complexes; 3) augmented concentrations of proteins mediating NLRP3 inflammasome activation such as nuclear factor-kappa B (NF-κB), NLRP3, apoptosis-associated speck-like protein containing a C-terminal caspase recruitment domain (ASC), and cleaved caspase-1; 4) heightened levels of end products resulting from NLRP3 inflammasome activation including interleukin (IL)-1β and IL-18; 5) enhanced leukotriene signaling; and 6) increased concentrations of oxidative stress markers malondialdehyde (MDA), protein carbonyls (PCs), as well as proinflammatory cytokines [28, 30, 31, 33]. Additionally, decreased neurogenesis within the hippocampus was observed throughout all time points studied following exposure to GWI-related chemicals up until 10 months post-exposure. Given that ongoing neurogenesis is known to contribute towards learning, memory, and mood regulation [34–37], diminished hippocampal neurogenesis likely serves as another contributing factor to cognitive and mood dysfunction seen in GWI.

Thus, strategies aimed at suppressing neuroinflammation and enhancing adult hippocampal neurogenesis are likely to have a positive impact on cognitive and mood function in veterans with chronic GWI. At present, there is no reliable treatment available for veterans with GWI that effectively minimizes chronic neuroinflammation, enhances neurogenesis, or improves cognitive and mood function [2]. In this context, cannabidiol (CBD) presents an attractive option for the treatment of GWI due to various reasons. CBD, a plant-based compound, is renowned for its non-psychological effects. It has been scientifically proven to possess anti-inflammatory and antioxidant properties, effectively inhibiting the signaling pathways involved in these processes [38–43]. Furthermore, CBD can inhibit glycogen synthase kinase-3β, which acts as a negative regulator of the Wnt/β-catenin pathway responsible for promoting neurogenesis in the hippocampus, an area of the brain involved in memory and learning [41, 44, 45]. Importantly, CBD has demonstrated an excellent safety profile even when taken long-term in low doses, with no observed harmful effects [46]. Epidiolex^®^ is a Food and Drug Administration (FDA)-approved CBD medication used for treating seizures associated with Lennox-Gastaut syndrome, Dravet syndrome, or tuberous sclerosis complex in patients aged one year or older [47]. The administration of CBD treatment began 6 months after exposure to chemicals related to GWI and stress. Therefore, this study investigated the efficacy of administering Epidiolex^®^ orally at a low-dose (20 mg/kg) over 16 weeks to enhance cognitive and mood function while reducing hyperalgesia in rats afflicted by chronic GWI.

## Methods

### Animals and study design

**Fig. 1:**
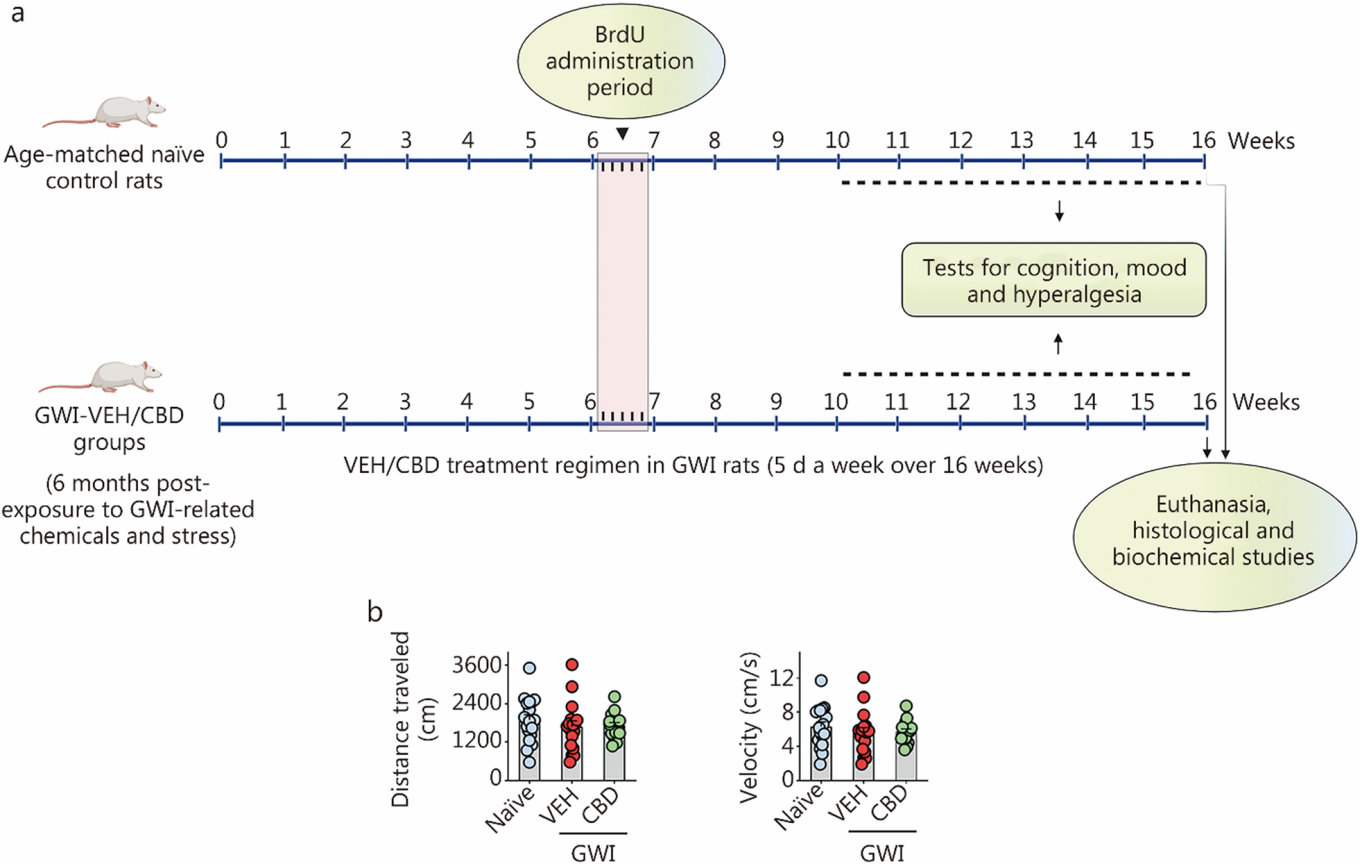
Timeline of experiments and the results of an open field test. **a** Schematic showing the study design, timelines of CBD treatment, neurocognitive studies, and brain tissue analyses. The rats were first exposed to Gulf War Illness (GWI)-related chemicals pyridostigmine bromide, DEET, and permethrin and 15 min of restraint stress for 28 d. Six months later, GWI rats received vehicle or CBD (20 mg/kg, 5 d/week) for 16 weeks until euthanasia. These rats received BrdU injections (i.p., 100 mg/kg) during the 7th week of CBD treatment. Starting from the 11th week after VEH/CBD treatment, animals were interrogated with a series of behavioral tests to ascertain cognition, mood, and pain. Age-matched naïve control rats received BrdU injections and were subjected to cognitive tests at matching time points as GWI rats receiving vehicle or CBD. The brain tissues were harvested for immunohistochemical and biochemical studies. CBD treatment did not impact motor function in rats with chronic GWI. **b** The bar charts compare the distances traveled and velocities of movement in an open field test across groups. Please refer to Table S3 in Additional file 1 for detailed statistical information. CBD cannabidiol, DEET N,N-diethyl-meta-toluamide, VEH Vehicle, BrdU 5’-bromodeoxyuridine

The rats used in this study were 8-week-old male Sprague Dawley rats (*n* = 53; Harlan, Indianapolis, IN, USA) and were provided with ad libitum access to standard chow containing 24.3% crude protein, 4.7% fat, 40.2% carbohydrates, and 4% crude fiber (Teklad rodent diet 8604; Inotiv, West Lafayette, Indiana, USA), as well as water. All studies conducted in this investigation received approval from the Institutional Animal Care and Use Committee of Texas A&M University (Animal Use Protocol number: 2022-0166D). The rats were randomly assigned to either the naïve control group (*n* = 18) or the GWI group (*n* = 35). A schematic diagram outlining the procedures and analyses conducted in this investigation is depicted in Fig. 1a. Rats in the GWI group received oral administration of PB, dermal application of DEET and PER, as well as daily restraint stress for 15 min for 28 d, following previously described protocols [28–31, 33]. Six months after exposure to chemicals and stress, a cohort of rats from the GWI group underwent vehicle (VEH, sterile water) treatment (GWI-VEH group; *n* = 17) for 16 weeks (5 d/week), while another cohort received Epidiolex^®^ treatment (GWI-CBD group; *n* = 18) during the same period (5 d/week). The decision to administer VEH or CBD to GWI rats starting from 6 months post-exposure was based on previous findings that at this time point these animals exhibit significant cognitive and mood impairments, along with increased oxidative stress and neuroinflammation [28]. A separate cohort of rats subjected solely to stress was not included in parallel, since an independent study conducted in our laboratory demonstrated that exposure to stress alone (i.e., 15 min of restraint stress daily for 28 d) does not lead to cognitive impairments, neuroinflammation or decline in hippocampal neurogenesis (see Additional file 1: Results and Figs. S1–S5).

During the 7th week of VEH/CBD treatment, the rats received 5’-bromodeoxyuridine (BrdU) injections (100 mg/kg) for 5 d to label newly generated cells and neurons in the hippocampus. Starting from the 11th week after VEH/CBD treatment, animals in all groups were tested with a series of neurobehavioral tasks during the daytime. After 16 weeks of VEH/CBD treatment, subgroups of rats were deeply anesthetized using isoflurane, and their brains were fixed with 4% paraformaldehyde via intracardiac perfusions. The fixed brain tissues were then processed for immunohistochemical and immunofluorescence analyses. Additional subgroups of rats were deeply anesthetized using isoflurane followed by decapitation. Fresh brains were harvested, snap-frozen, and stored at − 80 °C until used for biochemical assays. The investigators who performed the neurobehavioral tests, immunohistochemical or immunofluorescence quantifications, as well as biochemical assays remained blinded to group identification of animals or samples throughout the study period.

#### Exposures to GWI-related chemicals and restraint stress

The dosages administered were 2 mg/kg of PB in 0.5 ml of sterile water, 60 mg/kg of DEET in 0.2 ml of 70% ethanol, and 0.2 mg/kg of PER in 0.2 ml of a solution containing 70% ethanol, and 15 min of restrained stress for 28 d continuously. The application sites for both DEET and PER were on the skin along the dorsal surface of the neck and between scapulae regions respectively. Age-matched naïve control animals were maintained in parallel. The details of the chemicals are listed in Additional file 1: Table S1.

#### CBD treatment regimen in GWI rats

A liquid formulation of CBD (Epidiolex^®^), obtained from Jazz Pharmaceuticals (Philadelphia, PA, USA), was suspended in 0.5 ml of sterile water. Animals in the GWI-VEH group received 0.5 ml of sterile water. The dose of CBD is determined based on previous studies that have tested the efficacy of CBD in both naïve animals and animal models of neurodegenerative diseases [48–53]. Previous research has reported that administration of CBD to naïve control animals at doses ranging from 10 to 20 mg/(kg·d) does not result in any observable physiological changes [48, 49]. A recent study demonstrated that 3-month-old naïve control male and female mice receiving CBD [20 mg/(kg·d)] for over 14 weeks did not lead to alterations in spatial and novel object (NO) recognition memory functions, locomotor activity, mood function, or hippocampal neuron survival [48]. Furthermore, in neurodegenerative disease models, a dose of 20 mg/kg was found to be beneficial [50]. Additionally, animal models mimicking Alzheimer’s disease showed reduced neuroinflammation [51], increased neurogenesis [52], and improved cognitive and social interaction abilities [53] when administered at doses ranging from 10 to 20 mg/kg. Therefore, we chose to investigate a lower dose of CBD [20 mg/(kg·d)] in rats with chronic GWI. According to the formula suggested by Nair and Jacob [54], the human equivalent dosage would be approximately 3.24 mg/(kg·d). Thus, for an individual weighing 80 kg, this would translate to a daily dose of approximately 260 mg of CBD.

### Analyses of motor, cognitive, and mood function and nociception in GWI rats

Changes in motor function were evaluated in animals from all groups using an open field test (OFT). Then, 5 neurobehavioral tests were employed to investigate the cognitive and mood function as well as nociception. An object-in-place test (OIPT) was used to test the ability of animals to associate objects with previously encountered locations [55]. Proficiency in this task relies on an interaction between the hippocampus, the perirhinal cortex, and the medial prefrontal cortex. An object location test (OLT) was utilized to assess the capacity of animals to discern subtle changes in their immediate environment, a cognitive function dependent on the integrity of the hippocampus [55]. A pattern separation test (PST) examined the ability of animals to discriminate similar but not identical representations in a non-overlapping manner. Maintenance of this function depends upon the integrity of the dentate gyrus (DG) in the hippocampus, including the extent of neurogenesis [56, 57]. The degree of anhedonia, a depressive-like behavior, was evaluated through a sucrose preference test (SPT) [58, 59]. Hyperalgesia was ascertained using an electric Von Frey apparatus (Maze Engineers), which measured the force [millinewtons (mN)] exerted by the monofilament applied perpendicularly to the paw with an incremental increase until eliciting the paw withdrawal reflex [60].

#### Evaluation of motor function using an OFT

The rats from different groups were individually placed in the center of a brightly lit open field apparatus (100 cm × 100 cm Plexiglas open field box). Their movements were recorded using the Any-maze video tracking system for 5 min to measure both total distance traveled and mean velocity.

#### Evaluation of associative recognition memory using an OIPT

**Fig. 2:**
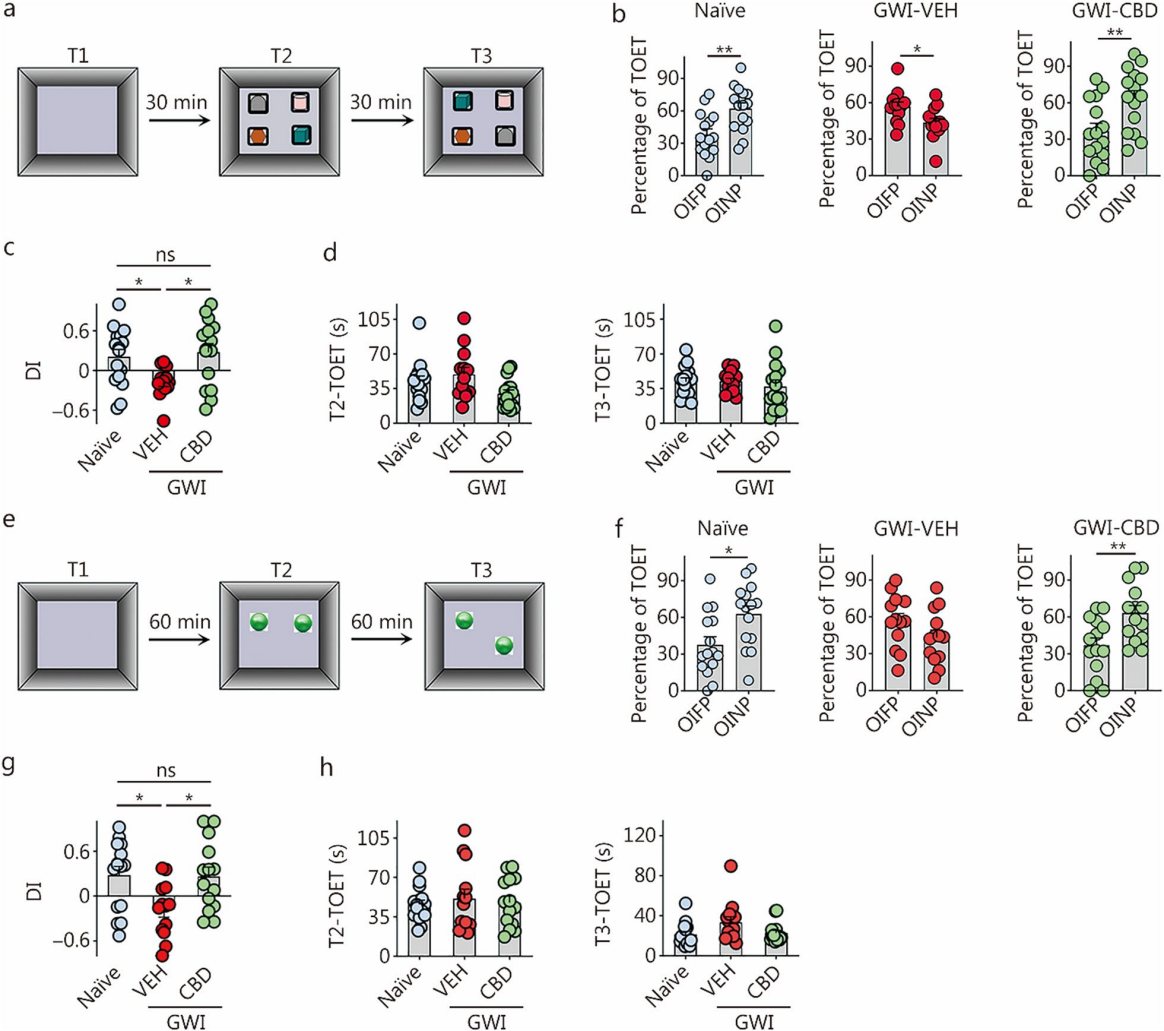
CBD treatment enhanced associative recognition and object location memory in rats with chronic GWI. **a** The cartoon illustrates the sequence of trials in an OIPT. **b** The bar charts compare percentages of the TOETs spent with the OIFP and the OINP in different animal groups (naïve, *n* = 15; GWI-VEH, *n* = 13; GWI-CBD, *n* = 15). **c** The bar chart compares the OINP-DI values across groups. **d** The bar charts compare the TOETs across groups in T2 and T3. **e** The cartoon illustrates the sequence of trials in an OLT. **f** The bar charts compare percentages of the TOETs spent with the OIFP and OINP in different animal groups (naïve, *n* = 15; GWI-VEH, *n* = 13; GWI-CBD, *n* = 14). **g** The bar chart compares the OINP-DI values across groups. **h** The bar charts compare the TOETs across groups in T2 and T3. ^*^P < 0.05, ^**^P < 0.01, ns non-significant. Please refer to Tables S3 and S4 in Additional file 1 for detailed statistical information. CBD cannabidiol, GWI Gulf War Illness, OIPT object-in-place test, OIFP objects in familiar place, OINP objects in novel place, VEH vehicle, DI discrimination index, TOET total object exploration time, T trial, OLT object location test

Each group of animals (*n* = 14–18) underwent 3 trials, namely trials 1–3 (T1–T3). In T1, the animals explored an empty open field apparatus for 5 min. In T2, which began 30 min after T1, 4 different objects were placed in 4 quadrants of the open field apparatus, and the animals explored them for 5 min (Fig. 2a). In T3, which started another 30 min later, the animals were placed in the middle of the open field apparatus with two objects from T2 remaining in their original locations and the other two objects from T2 swapping their sites diagonally (Fig. 2a). The behavior of each rat in T2 and T3 was video-tracked using the Any-maze video tracking system to obtain results such as times spent with objects in familiar places (OIFP), objects in novel places (OINP, for swapped objects) and total object exploration times (TOETs) for T2 and T3. Within each group, percentages of times spent exploring OIFP vs. OINP were statistically evaluated. Only data from animals that explored all 4 distinct objects carefully in at least 16 s during T2 and 8 s during T3 were included for analysis (Fig. 2a). Most groups met the criteria with 13–15 rats per group.

#### Investigation of object location memory using an OLT

Animals (*n* = 17–18) were subjected to 3 trials (T1–T3). During T1, the animals were allowed to explore an empty open field apparatus for 5 min. In T2, which began 60 min after T1, the animals explored 2 identical objects placed in 2 quadrants of the open field apparatus for another 5 min (Fig. 2e). T3 commenced 60 min later, and during this trial, the animals were positioned in the center of the open field apparatus, with one object still in its original location from T2 and the other object being relocated to a new position (Fig. 2e). The behavior of each rat during T2 and T3 was monitored using the Any-maze video tracking system. The resulting data include the times spent with OIFP compared to OINP, as well as TOETs for T2 and T3. Subsequently, we calculated the percentage of TOETs spent with OINP relative to OIFP within each group. Since proficiency in this test relies on thorough exploration of two distinct objects during T2 (Fig. 2e), only animals that explored objects for at least 16 s in T2 and at least 8 s in T3 were included for data analysis. Most groups consisted of between 13 and 15 rats that met these criteria.

#### Assessment of pattern separation ability using a PST

**Fig. 3:**
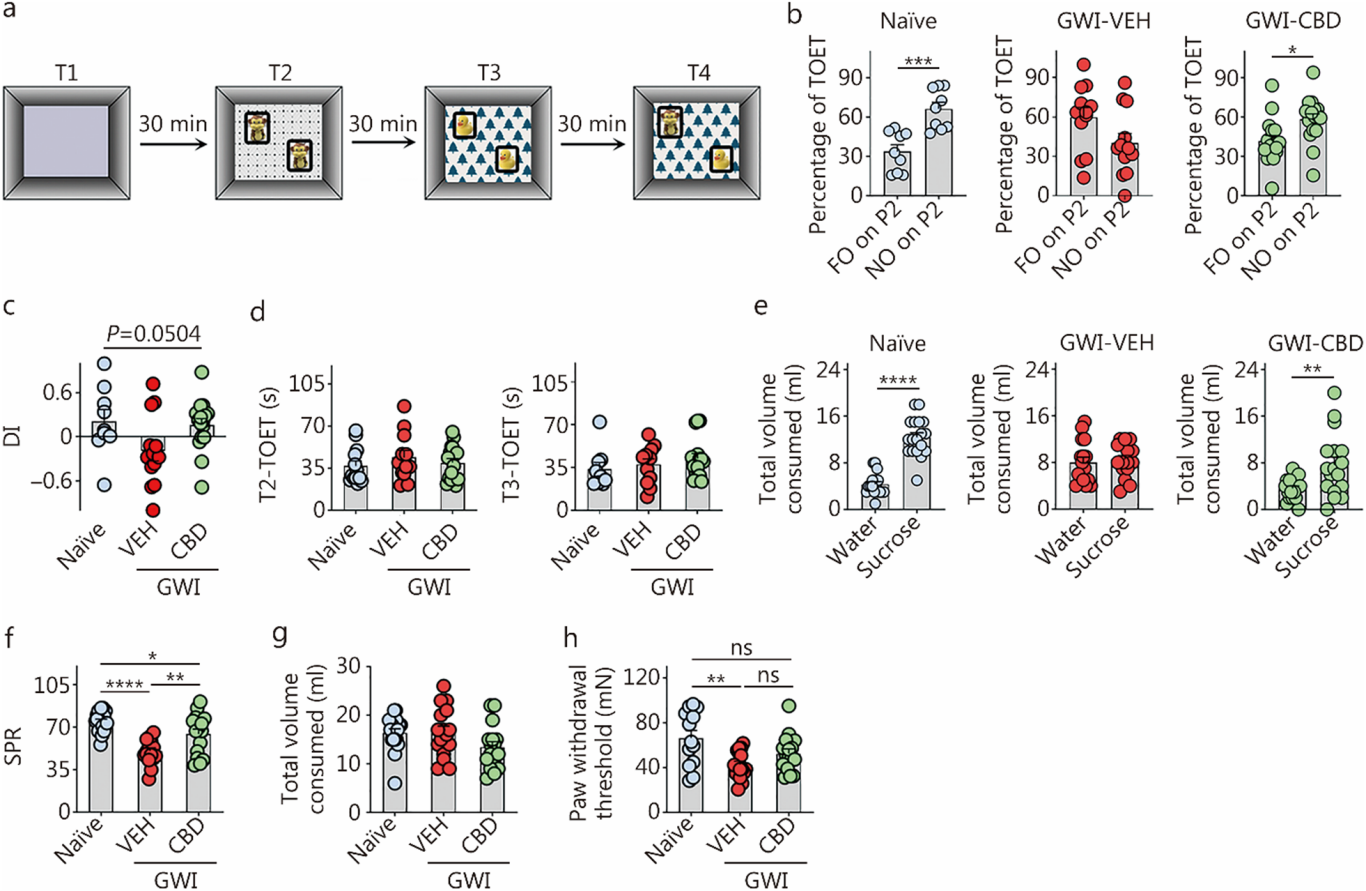
CBD treatment enhanced pattern separation function and reduced anhedonia and hyperalgesia in rats with chronic GWI. **a** The cartoon illustrates the sequence of trials in PST. **b** The bar charts compare percentages of the object exploration times spent with the FO on P2 and the NO on P2 in different groups (naïve, *n* = 10; GWI-VEH, *n* = 12; GWI-CBD, *n* = 16). **c** The bar chart compares the NO on P2 DI values across groups. **d** The bar charts compare the TOETs across groups in T2 and T3. **e** The bar charts illustrate the volumes of water and sucrose consumed by animals in naïve control, GWI-VEH, and GWI-CBD groups in the sucrose preference test. The bar charts show the SPR (**f**) and total volume consumed (**g**). **h** The bar graph compares the paw withdrawal threshold values across groups from a hyperalgesia test. ^*^P < 0.05, ^**^P < 0.01, ^***^P < 0.001, ^****^P < 0.0001, ns non-significant. Please refer to Tables S3 and S4 in Additional file 1 for detailed statistical information. CBD cannabidiol, GWI Gulf War Illness, PST pattern separation test, FO on P2 familiar object on pattern 2, NO on P2 novel object on pattern 2, VEH vehicle, TOET total object exploration time, T Trial, SPR sucrose preference rate, mN millinewtons, DI discrimination index

Animals (*n* = 13–17) underwent 4 trials (T1–T4). Following the exploration of the open field apparatus for 8 min (T1), each rat successively explored 2 distinct sets of identical objects (object types 1 and 2) placed on 2 different floor patterns (patterns 1 and 2, referred to as P1 and P2) for 8 min each in the 2 acquisition trials (T2–T3), with a separation interval of 30 min. Thirty minutes later, in T4, each rat explored one familiar object (FO) from T3 and one NO from T2, both placed on P2. The preference shown by the animals to explore the NO on P2 for longer durations compared to the FO on P2 reflects their ability to distinguish similar but not identical experiences. To ensure test validity, it was necessary for animals to thoroughly explore the location of distinct objects on both P1 and P2 during T2–T3 (Fig. 3a). Only animals that met the criteria by exploring objects for at least 20 s during these phases were included in data analysis. The majority of animals in each group (*n* = 9–16) fulfilled these criteria.

#### Evaluation of anhedonia using an SPT

Detailed methodology regarding the SPT can be found in our previous reports [30, 31]. In brief, the animal was acclimatized to 2 bottles on day 1, each with sucrose-containing water (sweet water). On day 2, the animal had access to sweet and standard water. On day 3, following a period of food and water deprivation lasting for 22 h, the animal was once again provided with access to sweet and standard water.

#### Evaluation of hyperalgesia using the Von Frey test

The Von Frey apparatus was employed to quantify the perpendicular force (mN) exerted by a monofilament on the paw of each animal. The paw withdrawal thresholds were then calculated as an indicator of mechanical allodynia and hyperalgesia in different animals [60].

### Tissue processing and immunohistochemistry

The fixed brain tissues were processed for cryostat sectioning, and 30-µm thick coronal brain tissue sections were stored at − 20 °C in a cryobuffer until further use, as described in our previous reports [28–31, 61–63]. Serial sections (every 15th or 20th) through the septotemporal axis of the entire hippocampus from each animal were processed for immunohistochemical studies to identify glial fibrillary acidic protein (GFAP)^+^ astrocytes, ionized calcium-binding adapter molecule-1 (IBA-1)^+^ microglia, newly born cells and neurons expressing BrdU or doublecortin (DCX). The primary and secondary antibodies employed are listed in Additional file 1: Table S2. The avidin–biotin complex reagent and the 3,3′-diaminobenzidine and Vector Gray substrate kits were supplied by Vector Labs (Burlingame, CA, USA). The brain tissue sections were mounted on gelatin-coated slides, air-dried, counterstained with nuclear fast red or hematoxylin (Vector Labs), dehydrated, cleared, coverslipped with permount solution, and examined under a Nikon E600 microscope [28–31].

### Evaluation of activated microglia

A dual immunofluorescence technique was utilized to visualize microglia expressing both IBA-1 (a marker of all microglia) and cluster of differentiation 68 (CD68, a marker for activated microglia) in various regions of the hippocampus, following the methods described in our previous publications [17, 28, 30, 31]. The primary and secondary antibodies employed for IBA-1 and CD68 immunofluorescence studies are listed in Additional file 1: Table S2. Z-section analysis was performed using a Nikon confocal microscope to quantify the percentages of microglia expressing IBA-1 and CD68. We counted the total numbers of microglia as well as the percentages of microglia expressing CD68 per unit area (approximately 216 μm^2^), which were then converted into percentages representing microglia expression of CD68. For these measurements, three consecutive sections (every 20th section) were analyzed in each animal (*n* = 6) [17, 18].

### Quantification of IBA-1^+^ microglial elements

The area fractions (AFs) of microglia (IBA-1^+^ structures) were quantified in the DG and hippocampal CA1 and CA3 subfields (*n* = 6) using ImageJ. The AF of thresholded pixels was expressed as a percentage of the total image area, which was measured in square millimeters. The microscopic images from different hippocampal subfields were digitized using a Nikon E600 microscope equipped with a 20 × objective lens and connected to a digital video camera linked to a computer. Each grayscale image was imported into ImageJ, where a binary image was generated by selecting an appropriate threshold value that highlighted all IBA1^+^ immunostained elements while eliminating background noise. The area occupied by the IBA1^+^ structures (AF) in the binary image was then measured using the Analyze command within the software. The AF represents the proportion of highlighted pixels in the image. For each animal, separate calculations were performed to determine the AF of IBA1^+^ structures in each hippocampal region, using data from all chosen serial sections before determining the means and standard errors for each group (*n* = 6). Three serial sections (every 20th section) were analyzed per animal (*n* = 6) for these measurements [17, 18].

### Quantification of NLRP3 inflammasome complex in microglia

We next probed the formation of NLRP3 inflammasome complexes in microglia through a triple immunofluorescence method for NLRP3, ASC, and IBA-1 [30, 31, 63, 64]. The primary and secondary antibodies employed for NLRP3, ASC, and IBA-1 triple immunofluorescence studies are listed in Additional file 1: Table S2. The sections were next examined through 2-μm thick, Z-section analysis using Leica THUNDER 3D Imager. The total number of NLRP3 inflammasome complexes (i.e., structures displaying NLRP3 and ASC co-expression) per unit area (about 216 μm^2^) of the CA3 subfield was measured using three representative sections per animal (*n* = 5). Also, the percentages of IBA-1^+^ microglia containing NLRP3-ASC complexes were measured.

### Preparation of hippocampal tissue lysates for biochemical assays

Each hippocampus was microdissected and lysed by sonication for 15–20 s at 4 °C in a tissue extraction reagent containing protease and phosphatase inhibitor. Following centrifugation of the resulting solution for 10 min at 15,000*g*, the supernatant was aliquoted and kept at − 80 °C until further use. The details of the reagents are listed in Additional file 1: Table S1.

### Quantification of markers of the NLRP3 inflammasome pathway, the oxidative stress, the JAK/STAT pathway, anandamide, and astrocyte hypertrophy

The hippocampal lysates from rats belonging to different groups were processed to quantify the concentrations of various markers associated with NLRP3 inflammasome activation and proinflammatory cytokines (*n* = 6). Individual enzyme-linked immunoassay (ELISA) kits, as detailed in our previous studies [30, 64] and Additional file 1: Table S1, were used for this purpose.

The hippocampal lysates were also processed to measure the levels of MDA, PCs, catalase (CAT), and superoxide dismutase (SOD) using assay kits as detailed in our previous studies [30, 31] and Additional file 1: Table S1. The concentrations of these markers were normalized to the total protein content in the respective tissue lysates. Subsequently, we employed ELISA to quantify the mediators and markers associated with JAK/STAT signaling, including IL-6, interferon-γ (IFN-γ), phosphorylated JAK-1 (p-JAK-1), and phosphorylated STAT1 (p-STAT1). Additionally, we assayed the concentration of endogenous cannabinoid anandamide using a kit provided by MyBioSource (San Diego, CA, USA). The assay kits employed for these measurements are listed in Additional file 1: Table S1.

The AFs of astrocytes (GFAP^+^ structures) were determined in the DG and hippocampal CA1 and CA3 subfields (*n* = 6) using ImageJ. Three serial sections (every 20th) were employed in each animal (*n* = 6) for these measurements [17, 18].

### Measurement of newly born cells positive for BrdU and newly born neurons expressing DCX

The entire hippocampus was sectioned into 30-µm slices (every 15th slice) for visualization of BrdU^+^ newly born cells or DCX^+^ newly born neurons in the subgranular zone-granule cell layer (SGZ-GCL) of the hippocampus. A total of 6 sections were analyzed per marker per animal, with a sample size of *n* = 5–6. Stereological quantification of BrdU^+^ cells and DCX^+^ neurons was performed using a StereoInvestigator system (Microbrightfield, Williston, Vermont, USA), following the methodology described in our previous reports [31, 59, 65, 66]. Within each animal, BrdU^+^ cells and DCX^+^ neurons were counted from randomly and systematically selected frames measuring 40 μm × 40 μm (0.0016 mm^2^ area) in each chosen section under a 100 × oil immersion objective lens. The selection frequency of serial sections across groups was determined based on the density distribution of BrdU^+^ cells and DCX^+^ neurons, with every 15th section being included. The StereoInvestigator system’s optical fractionator component used a systematic random sampling scheme to determine the number and density of frames for analysis in each section. The dissector height for counting frames was set at 8 μm to ensure accurate cell counting. By selecting frames randomly and systematically throughout the SGZ-GCL in each serial section, we ensured that every BrdU^+^ cell or DCX^+^ neuron had an equal chance of being counted despite any uneven distribution within the SGZ-GCL among different experimental groups. The average Gunderson coefficient error values ranged from 0.126 to 0.198 for BrdU^+^ cells across all groups, and between 0.111 and 0.156 for DCX^+^ neurons.

### Quantification of neuronal differentiation of BrdU^+^ newly born cells, and net hippocampal neurogenesis

We subsequently quantified the percentages of newly born cells (i.e., BrdU^+^ cells) that underwent neuronal differentiation in the SGZ-GCL using dual immunofluorescence for BrdU and neuron-specific nuclear antigen (NeuN), as well as Z-section analyses [28, 31]. For these investigations, we utilized mouse anti-NeuN, mouse anti-BrdU, donkey anti-mouse IgG labeled with Alexa Flour 488, and donkey anti-rat IgG labeled with Alexa Flour 594. The primary and secondary antibodies employed for BrdU immunostaining and BrdU-NeuN dual immunofluorescence are listed in Additional file 1: Table S2. Two-micrometer thick optical Z-sections were taken using a Nikon confocal microscope from 3 representative sections at anterior, mid, and posterior levels of the hippocampus in each animal (*n* = 5, 25–30 BrdU^+^ cells/animal). Subsequently, by combining the absolute numbers of BrdU^+^ cells in the SGZ-GCL obtained through stereology with the percentages of BrdU^+^ cells expressing NeuN, we estimated the total number of mature neurons added to the GCL over 5 d in each animal.

### Statistical analyses

Data were expressed as means ± SEM. For comparing the two groups, either a two-tailed, unpaired Student’s *t*-test or the Mann–Whitney *U* test was employed, depending on the presence of significant differences in standard deviations between the groups. When dealing with comparisons involving three or more datasets, one-way analysis of variance (ANOVA), followed by Newman–Keuls multiple comparison post hoc tests, was used. In case any individual group failed the normality test (Shapiro–Wilk test), the Kruskal–Wallis test with Dunn’s post-hoc tests were utilized. To compare body weights across groups over 16 weeks, two-way repeated measures ANOVA was employed. Throughout all comparisons, statistical significance was defined as *P* < 0.05.

## Results

### CBD treatment did not cause weight loss in GWI rats

We monitored the body weights of GWI rats receiving either VEH or CBD throughout a 16-week treatment regimen, weekly. Throughout the entire 16-week treatment period, we did not observe any statistically significant differences in body weights between the two groups (Additional file 1: Fig. S6). Furthermore, no adverse effects were observed in GWI rats receiving CBD.

### CBD treatment did not impact motor function in GWI rats

Evaluation of the total distance traveled and the velocities of movement in an OFT revealed no differences in motor function among the naïve, GWI-VEH, and GWI-CBD groups (Fig. 1b). Thus, long-term CBD treatment did not induce motor impairments.

### CBD treatment improved associative recognition memory function in GWI rats

Naïve control rats demonstrated the ability to associate objects with specific locations based on previous encounters. This was evident from their preference to explore the 2 objects that swapped positions in T3 (i.e., OINP) over the 2 objects that remained in their original locations from T2 (OIFP; *P* < 0.01, Fig. 2b). Rats in the GWI-VEH group spent more time exploring OIFP during TOETs compared to OINP (*P* < 0.05, Fig. 2b), implying impaired recognition memory associated with spatial information. In contrast, rats in the GWI-CBD group allocated significantly higher percentage of their TOET towards the objects that swapped positions (OINP; *P* < 0.01, Fig. 2b), confirming intact spatial recognition memory. Comparison of the discrimination index (DI) for OINP using one-way ANOVA across groups revealed significant differences (Fig. 2c). The OINP-DI was significantly lower in the GWI-VEH group compared to the naïve control group (*P* < 0.05, Fig. 2c). Conversely, GWI rats receiving CBD displayed similar OINP-DI as observed in the naïve control group (*P* > 0.05, Fig. 2c). The duration of TOETs during T2 and T3 did not differ significantly between groups (*P* > 0.05, Fig. 2d). Thus, CBD treatment improved associative recognition memory function in GWI rats.

### CBD treatment alleviated object location memory dysfunction in GWI rats

In this test, animals proficient in object location memory show a greater inclination to inspect the object that has been relocated to a novel location (OINP) instead of the OIFP. In T3, naïve rats preferred the OINP over the OIFP (*P* < 0.05), whereas GWI rats treated with VEH showed impairment as they spent similar amounts of time exploring both the OIFP and OINP (*P* > 0.05, Fig. 2f). Conversely, GWI rats that received CBD exhibited increased percentages of their TOET with the OINP (*P* < 0.01, Fig. 2f), mirroring the behavior of naïve control rats. One-way ANOVA analysis of the OINP-DI values across groups confirmed the proficiency of the GWI-CBD group for object location memory, as their DI values matched those of animals in the naïve control group (*P* > 0.05, Fig. 2g). However, the DI values in the GWI-VEH group were lower than those of animals in both naïve and GWI-CBD groups (*P* < 0.05, Fig. 2g). The TOETs in T2 and T3 were comparable among all groups (*P* > 0.05, Fig. 2h). Thus, CBD treatment alleviated dysfunction in object location memory observed in GWI rats.

### CBD treatment eased pattern separation memory dysfunction in GWI rats

In the PST, animals proficient in pattern separation exhibited a greater preference for exploring the NO on P2 compared to the FO on P2 in T4. Impaired pattern separation was observed in rats belonging to the GWI-VEH group, as they did not show a significant preference for NO on P2 over FO on P2 (*P* > 0.05), unlike the naïve control group which demonstrated a strong preference for NO on P2 (*P* < 0.001, Fig. 3b). In contrast, rats in the GWI-CBD group spent a higher percentage of their TOET with NO on P2 (*P* < 0.05, Fig. 3b), exhibiting behavior similar to that of rats in the naïve control group. Differences between groups were also evident from the one-way ANOVA analysis of the DI values for the NO on P2 across groups (*P* = 0.05, Fig. 3c). The TOETs in T2 and T3 showed no significant differences among groups (*P* > 0.05, Fig. 3d). Thus, CBD treatment effectively improved pattern separation ability in GWI rats.

### CBD treatment alleviated anhedonia in GWI rats

Naïve control rats preferred sucrose water over standard water, indicating the absence of anhedonia (*P* < 0.0001, Fig. 3e). Rats in the GWI-VEH group displayed anhedonia, as evidenced by their equal preference for sucrose-containing water and regular water (*P* > 0.05, Fig. 3e). Remarkably, GWI rats treated with CBD exhibited a preference for sucrose-containing water compared to standard water, suggesting the absence of anhedonia (*P* < 0.01, Fig. 3e). The sucrose preference rate (SPR) was reduced in GWI rats that received VEH compared to naïve control rats (50.26% vs. 74.55%, *P* < 0.0001; Fig. 3f). Additionally, GWI rats treated with CBD showed significantly improved SPR than GWI-VEH rats (64.18% vs. 50.26%, *P* < 0.01; Fig. 3f). However, the overall SPR in the GWI-CBD group was still lower than that in the naïve control group (*P* < 0.05, Fig. 3f). Furthermore, there were no significant differences in the total fluid consumption between different groups (*P* > 0.05, Fig. 3g). Thus, while CBD treatment in GWI rats resulted in an increase of 13.92% sucrose preference, it did not fully restore their sucrose preference to levels observed in the naïve control group. Nonetheless, CBD treatment effectively alleviated anhedonia or depressive-like behavior in rats with chronic GWI.

### CBD treatment reduced hyperalgesia in GWI rats

The presence of persistent pain has emerged as one of the most incapacitating symptoms associated with chronic GWI [67, 68]. In line with this, rats in the GWI-VEH group exhibited significantly reduced paw withdrawal thresholds compared to those in the naïve control group during the Von Frey test (*P* < 0.01, Fig. 3h). In contrast, rats in the GWI-CBD group demonstrated paw withdrawal thresholds matched the thresholds that were comparable to those observed in the naïve control group (*P* > 0.05, Fig. 3h). Consequently, CBD treatment effectively reduced hyperalgesia in chronic GWI rats.

### CBD treatment diminished microgliosis and activation of microglia in chronic GWI rats

**Fig. 4:**
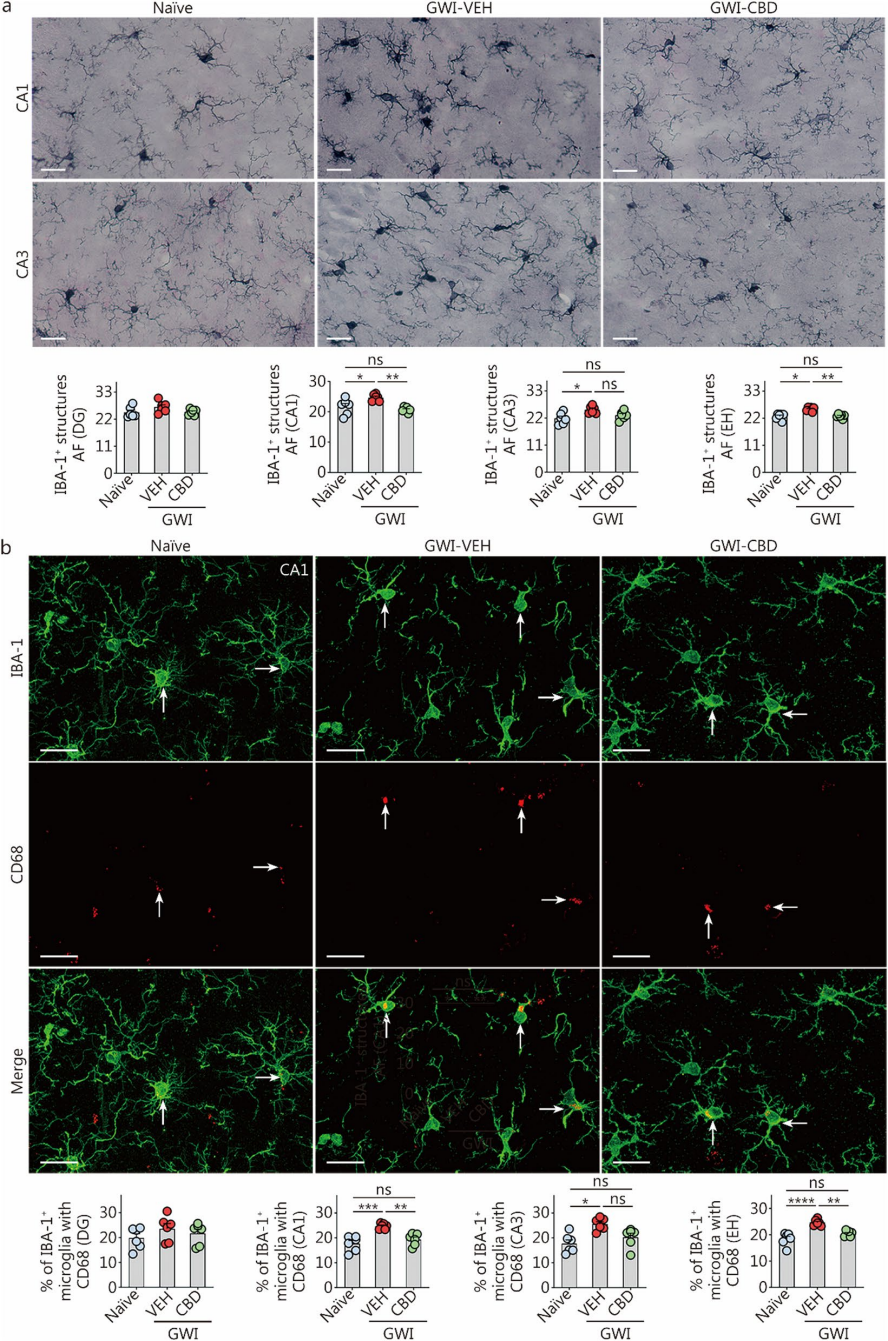
CBD treatment diminished microgliosis and the occurrence of proinflammatory microglia in rats with chronic GWI. **a** Figures illustrate examples of IBA-1^+^ microglia in the hippocampal CA1 and CA3 subfields from naïve control, GWI-VEH, and GWI-CBD groups (scale bar = 10 μm). The bar charts compare the AF of IBA-1^+^ microglial structures in the DG, CA1 subfield, CA3 subfield, and EH across groups. **b** Figures illustrate the examples of IBA-1^+^ microglia (green) expressing CD68 (red) in the hippocampal CA1 subfield for naïve control, GWI-VEH, and GWI-CBD groups (scale bar = 10 μm). Arrows indicate IBA-1^+^ microglia expressing CD68. The bar charts compare percentages of IBA-1^+^ microglia with CD68 in the DG, CA1 subfield, CA3 subfield, and EH across groups. ^*^P < 0.05, ^**^P < 0.01, ^***^P < 0.001, ^****^P < 0.0001, ns non-significant. Please refer to Table S3 in Additional file 1 for detailed statistical information. CBD cannabidiol, GWI Gulf War Illness, VEH vehicle, IBA-1 ionized calcium-binding adapter molecule-1, DG dentate gyrus, CA cornu ammonis, EH entire hippocampus, CD68 cluster of differentiation 68, AF area fraction

We utilized ImageJ to analyze the AF of IBA-1^+^ microglial elements in the hippocampus. Figure 4a depicts the IBA-1^+^ microglial structures in the hippocampal CA1 and CA3 subfields. One-way ANOVA revealed significant differences in AFs of IBA-1^+^ elements between groups in both CA1 and CA3 regions, as well as when considering the entire hippocampus (*P* < 0.01, Fig. 4a). However, no such differences were observed in the DG region (*P* > 0.05, Fig. 4a). In comparison to the naïve control group, higher levels of IBA-1^+^ microglial elements were observed in the GWI-VEH group within CA1, CA3 subfields, and throughout the entire hippocampus (*P* < 0.05, Fig. 4a). In contrast, CBD treatment in GWI rats normalized the extent of IBA-1^+^ microglial elements to those in the naïve control group (*P* > 0.05, Fig. 4a). The AF of IBA-1^+^ microglial elements was lower in both CA1 subfield and entire hippocampus for GWI-CBD group compared to the GWI-VEH group (*P* < 0.01, Fig. 4a). The increased density and hypertrophy of microglia soma likely contributed to higher AFs of microglial elements observed in the hippocampus of GWI-VEH group compared to naïve control group. Moreover, there was a comparable level of AFs between GWI-CBD and naïve control groups since CBD treatment reduced both density and hypertrophy of microglia soma. Qualitatively, microglial morphology resembled that seen in the naïve control group for GWI-CBD rats. Thus, CBD treatment effectively mitigated hyperplasia and hypertrophy exhibited by microglia soma in GWI rats’ brains.

We also examined the presence of activated microglia in the CA1, CA3, and DG subfields of the hippocampus (Fig. 4b). Activated microglia were visualized through dual immunofluorescence staining for IBA-1 and CD68. One-way ANOVA analysis revealed significant differences between groups in the CA1 and CA3 subfields as well as the entire hippocampus (*P* < 0.05, Fig. 4b). However, no such differences were observed in the DG subfield (*P* > 0.05, Fig. 4b). Post hoc tests conducted on the CA1 and CA3 subfields and the whole hippocampus uncovered that rats in the GWI-VEH group exhibited higher percentages of activated microglia compared to those in the naïve control group (*P* < 0.05, Fig. 4b). Treatment with CBD reduced these percentages of the IBA-1^+^ cells expressing CD68 to levels comparable to those seen in naïve controls (*P* > 0.05, Fig. 4b), implying mitigation of activated microglia phenotypes by CBD treatment. Notably, there was a significant reduction in percentages of activated microglia in both the CA1 subfield and the entire hippocampus following CBD treatment compared to the GWI-VEH group (*P* < 0.01, Fig. 4b). Thus, CBD treatment effectively decreased the percentages of activated microglia in the hippocampus.

### CBD treatment inhibited the activation of NLRP3 inflammasomes in chronic GWI rats

**Fig. 5:**
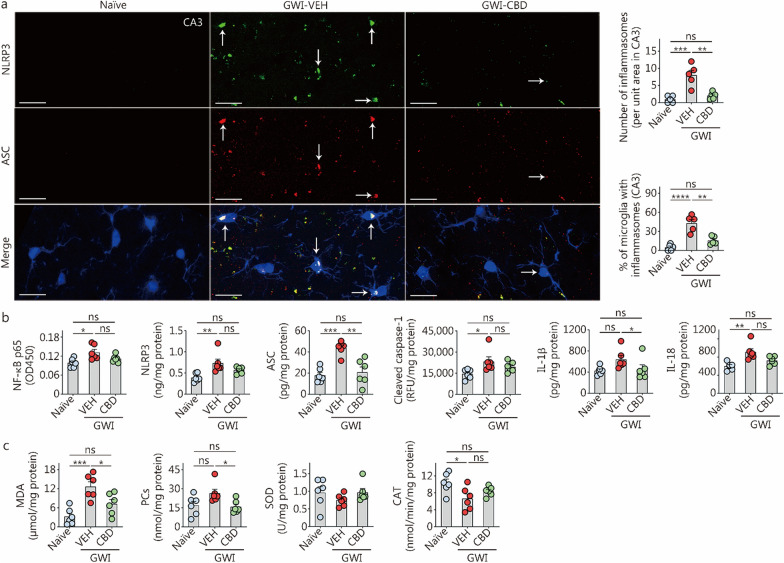
CBD treatment reduced the inflammasome activation and oxidative stress in the hippocampus of rats with chronic Gulf War Illness (GWI). **a** The image panels illustrate the examples of IBA1^+^ microglia (blue) displaying NLRP3 (green) and ASC (red) structures, and NLRP3-ASC complexes (whitish structures in merge) from naïve control, GWI-VEH, and GWI-CBD groups (scale bar = 20 μm). Arrows point to IBA-1^+^ microglia (blue) expressing NLRP3 (green) and ASC (red) and NLRP3^+^ ASC complexes (white). The yellowish structures are NLRP3-ASC inflammasome complexes outside the microglia, likely the inflammasomes in other cell types, such as astrocytes and neurons. The bar charts compare the number of inflammasomes per unit area in the CA3 subfield and percentages of microglia expressing inflammasomes across groups. **b** The bar charts compare the concentrations of the NF-κB p65, NLRP3, ASC, cleaved caspase-1, IL-1β, and IL-18 across naïve control, GWI-VEH and GWI-CBD groups. **c** The bar charts compare the concentrations of MDA, PCs, SOD, and CAT across groups. ^*^P < 0.05, ^**^P < 0.01, ^***^P < 0.001, ^****^P < 0.0001, ns non-significant. Please refer to Table S3 in Additional file 1 for detailed statistical information. CAT catalase, CBD cannabidiol, VEH vehicle, NF-κB nuclear factor-kappa B, NLRP3 NOD-, LRR- and pyrin domain-containing protein 3, ASC apoptosis-associated speck-like protein containing a C-terminal caspase recruitment domain, IL interleukin, MDA malondialdehyde, PCs protein carbonyls, SOD superoxide dismutase, RFU relative fluorescence unit

We investigated the formation of NLRP3 inflammasome complexes and their activation in the hippocampus. Using triple immunofluorescence staining for NLRP3, ASC, and IBA-1, we initially observed the activation of NLRP3 inflammasome complexes specifically in microglia (Fig. 5a). One-way ANOVA analysis uncovered significant differences between groups regarding both the number of inflammasome complexes per unit area and the percentages of microglia with inflammasome complexes (*P* < 0.001, Fig. 5a). Compared to the naïve control group, the GWI-VEH group exhibited an elevated number of inflammasome complexes as well as a higher percentage of microglia expressing inflammasome complexes (*P* < 0.001, Fig. 5a). Treatment with CBD resulted in a reduction in both the number of inflammasome complexes per unit area and the percentages of microglia expressing inflammasome complexes in the hippocampus (*P* < 0.01, Fig. 5a). Consequently, CBD treatment effectively decreased overall incidence rates of NLRP3 inflammasome complex formation within microglia located in the hippocampus.

Next, we quantified the concentrations of the transcription factor NF-κB p65 and mediators involved in NLRP3 signaling (NLRP3, ASC, and cleaved caspase-1) in the hippocampus. One-way ANOVA analysis revealed significant differences among groups for these markers (*P* < 0.05, Fig. 5b). The concentrations of these proteins were found to be upregulated in the GWI-VEH group compared to the naïve control group (*P* < 0.05, Fig. 5b). Notably, CBD treatment normalized the concentrations of these proteins to levels observed in the naïve control group (*P* > 0.05, Fig. 5b). Furthermore, there were significant variations between groups regarding IL-1β and IL-18, which are end products of NLRP3 inflammasome activation (*P* < 0.05, Fig. 5b). The concentration of IL-18 was significantly increased in the GWI-VEH group (*P* < 0.01, Fig. 5b) but it returned to a level similar to that seen in the naïve control with CBD treatment. Additionally, compared to GWI-VEH, GWI-CBD showed a lower concentration of IL-1β (*P* < 0.05, Fig. 5b). Overall, CBD treatment in GWI rats reduced concentrations of the transcription factor NF-κB p65 responsible for triggering the formation of NLRP3 inflammasome complexes and proteins that facilitate NLRP3 inflammasome activation (NLRP3, ASC, and cleaved caspase-1), leading to decreased levels of proinflammatory cytokines IL-1β and IL-18 in the microenvironment. Thus, CBD treatment effectively suppresses chronic inflammation perpetuated by NLRP3 inflammasome activation.

### CBD treatment diminished oxidative stress in chronic GWI rats

We investigated the efficacy of CBD in diminishing oxidative stress in rats with chronic GWI by quantifying concentrations of oxidative stress (MDA and PCs) and antioxidant markers (SOD and CAT). One-way ANOVA demonstrated significant differences between groups for MDA, PCs, and CAT (*P* < 0.05, Fig. 5c). However, no significant differences were observed between groups for SOD (*P* > 0.05, Fig. 5c). Post hoc analysis revealed that the concentration of MDA was significantly higher in the GWI-VEH group compared to the naïve control group (*P* < 0.001, Fig. 5c). Although not statistically significant, the concentration of PCs was also higher in the GWI-VEH group than in the naïve control group (*P* > 0.05, Fig. 5c). In contrast, both MDA and PCs concentrations were reduced in the GWI-CBD group compared to the GWI-VEH group (*P* < 0.05), resembling levels seen in the naïve control group (*P* > 0.05, Fig. 5c). The concentration of CAT was lower in the GWI-VEH group compared to the naïve control group (*P* < 0.05), while it remained similar to that of the naïve control group after CBD treatment in the GWI-CBD group (*P* > 0.05, Fig. 5c). Thus, CBD treatment reduced oxidative stress in rats with GWI.

### CBD treatment mitigated JAK/STAT signaling in chronic GWI rats

**Fig. 6:**
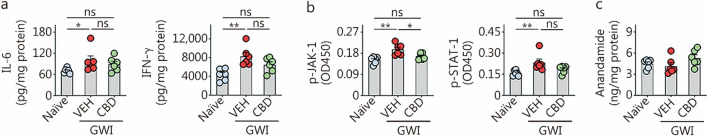
CBD treatment reduced the activation of Janus kinase/signal transducers and activators of transcription (JAK/STAT) pathway but did not affect the endogenous cannabinoid, anandamide in rats with chronic Gulf War Illness (GWI). **a**–**c** The bar charts compare the concentrations of IL-6, IFN-γ, p-JAK-1, p-STAT-1, and anandamide across groups. ^*^P < 0.05, ^**^P < 0.01, ns non-significant. Please refer to Table S3 in Additional file 1 for detailed statistical information. CBD cannabidiol, VEH vehicle, IFN-γ interferon-γ, p-JAK-1 phosphorylated Janus kinase-1, p-STAT-1 phosphorylated signal transducer and activator of transcription-1

We investigated whether CBD treatment can modulate the activation of JAK/STAT signaling in GWI rats by quantifying proteins that trigger (IL-6 and IFN-γ) and activate (p-JAK-1 and p-STAT-1) JAK/STAT pathway. One-way ANOVA analysis of IL-6 and IFN-γ concentrations across groups revealed significant differences between groups (*P* < 0.01, Fig. 6a). Notably, both IL-6 and IFN-γ were significantly upregulated in the hippocampus of the GWI-VEH group (*P* < 0.05, Fig. 6a). However, CBD treatment normalized the concentrations of IL-6 and IFN-γ to levels observed in the naïve control group (*P* > 0.05, Fig. 6a). The decreased levels of IL-6 and IFN-γ mediated by CBD led to reduced concentrations of p-JAK-1 and p-STAT-1 in the GWI-CBD group. While one-way ANOVA analysis revealed significant differences between groups for p-JAK-1 and p-STAT-1 (*P* < 0.01, Fig. 6b), post hoc analysis showed that concentrations of p-JAK-1 and p-STAT-1 were significantly upregulated in the hippocampus of the GWI-VEH group compared to those in the naïve control group (*P* < 0.05, Fig. 6b). Notably, CBD treatment normalized the concentrations of p-JAK-1 and p-STAT-1 in the GWI-CBD group to levels in the naïve control group (*P* > 0.05, Fig. 6b). Thus, CBD treatment can suppress the activation of the JAK/STAT signaling in GWI rats by reducing the concentrations of IL-6 and IFN-γ.

### CBD treatment did not alter endogenous anandamide levels in chronic GWI rats

The effect of CBD treatment on endogenous cannabinoid N-arachidonoylethanolamine (or anandamide) in chronic GWI was also investigated. One-way ANOVA analysis did not reveal any significant differences between the groups (*P* > 0.05, Fig. 6c), suggesting that exogenous CBD did not affect the concentration of endogenous cannabinoids in chronic GWI.

### CBD treatment diminished hypertrophy of astrocytes in chronic GWI rats

**Fig. 7:**
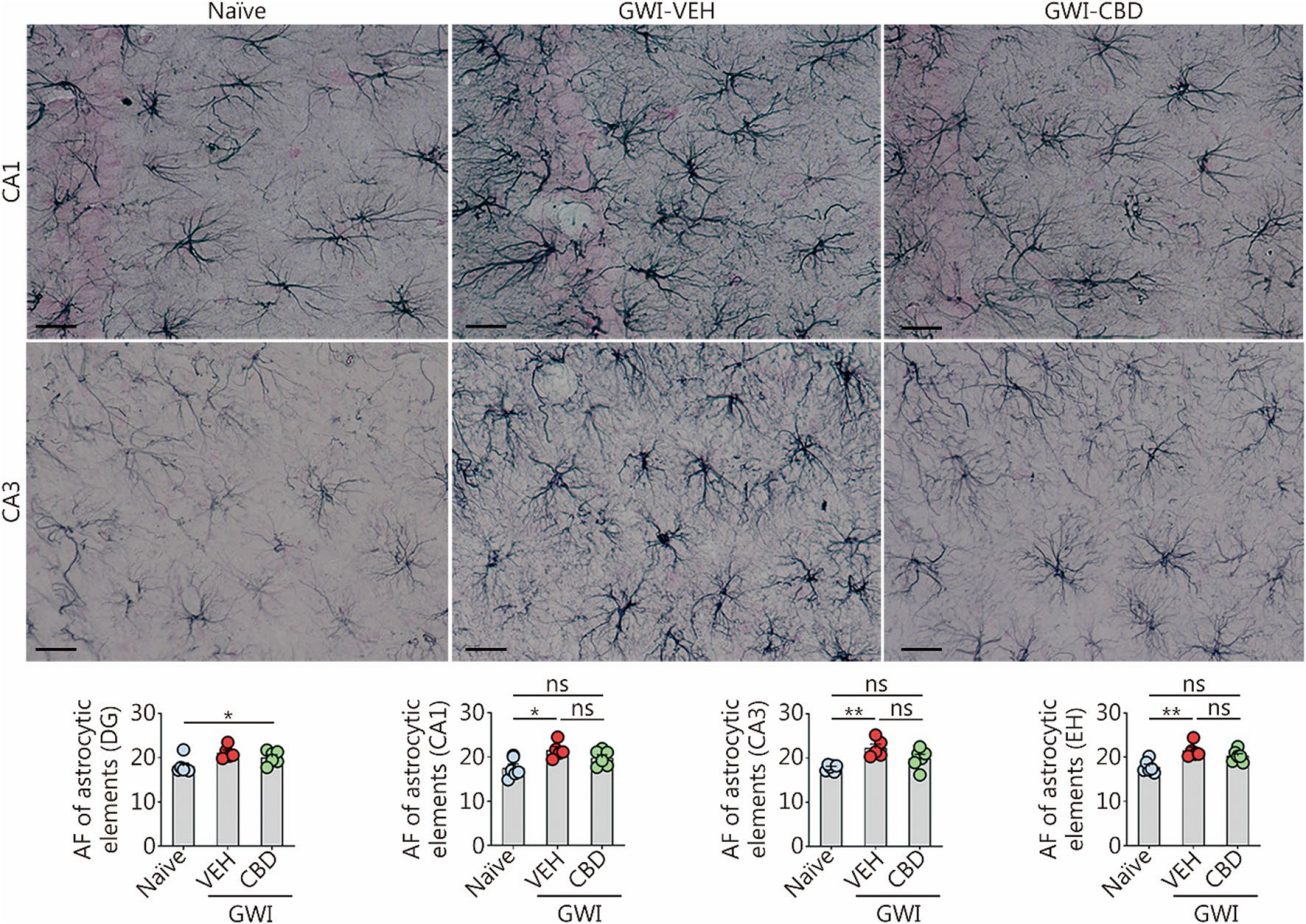
CBD treatment reduced astrocyte hypertrophy in the hippocampus of rats with chronic GWI. Figures illustrate examples of GFAP^+^ astrocytes in the hippocampal CA1 and CA3 subfields from naïve control, GWI-VEH, and GWI-CBD groups (scale bar = 25 μm). The bar charts compare the AF of astrocytic elements in the DG, CA1 subfield, CA3 subfield, and EH across groups. ^*^P < 0.05, ^**^P < 0.01, ns non-significant. Please refer to Table S3 in Additional file 1 for detailed statistical information. CBD cannabidiol, GWI Gulf War Illness, GFAP glial fibrillary acidic protein, VEH vehicle, AF area fraction, DG dentate gyrus, CA cornu ammonis, EH entire hippocampus

Using ImageJ, we examined the AF of GFAP^+^ astrocytes in different subfields of the hippocampus, including DG, CA1, and CA3. The representative images displayed GFAP^+^ astrocytes specifically in the CA1 and CA3 subregions of the hippocampus (Fig. 7). Statistical analysis using one-way ANOVA revealed significant differences among groups regarding GFAP^+^ structures within these regions (*P* < 0.05, Fig. 7). Subsequent post hoc analysis demonstrated that compared to the naïve control group, GWI-VEH group exhibited elevated levels of GFAP^+^ structures in both CA1 and CA3 subfields as well as throughout the entire hippocampus (*P* < 0.05, Fig. 7). Although ANOVA analysis indicated statistical differences between groups in DG, no significant variations were observed through post hoc tests (Fig. 7). Notably, CBD treatment normalized astrocyte features as evidenced by comparable levels of astrocytic elements between GWI-CBD group and naïve control group within these regions (*P* > 0.05, Fig. 7). Thereby, CBD treatment reduced astrocyte hypertrophy in the hippocampus of GWI rats.

### CBD treatment improved hippocampal neurogenesis in chronic GWI rats

**Fig. 8:**
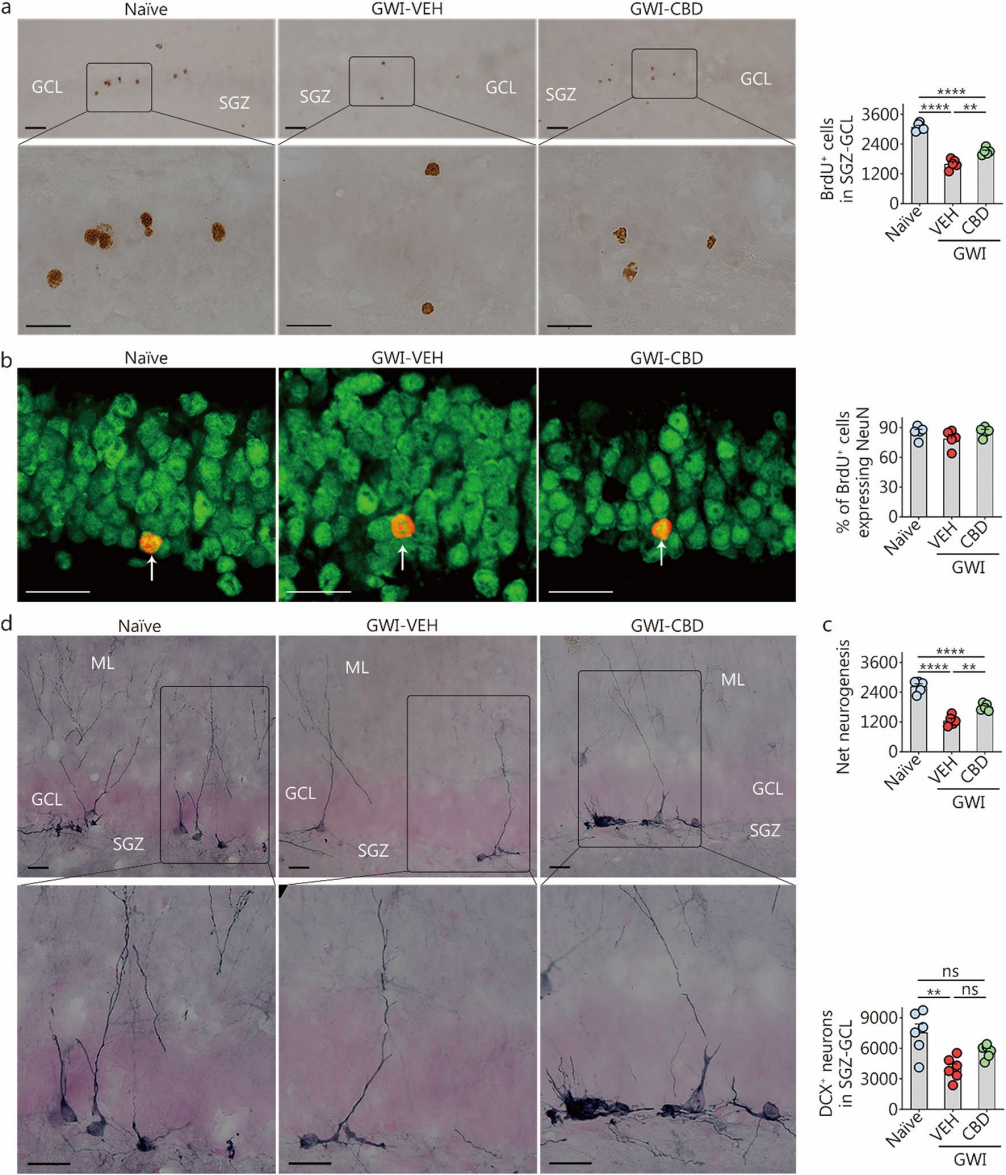
CBD treatment enhanced net hippocampal neurogenesis and the status of hippocampal neurogenesis in rats with chronic Gulf War Illness (GWI). **a** Figures illustrate examples of BrdU^+^ newly born cells in the SGZ-GCL of the hippocampus from naïve control, GWI-VEH, and GWI-CBD groups (scale bar = 25 µm). The bar chart compares the number of BrdU^+^ newly born cells in the SGZ-GCL of the hippocampus across groups. **b** Figures illustrate examples of BrdU^+^ cells expressing the neuronal marker, NeuN (arrows) from naïve control, GWI-VEH, and GWI-CBD groups (scale bar = 25 µm). Arrows denote BrdU^+^ cells expressing the neuronal marker NeuN. Bar charts compare percentages of BrdU^+^ cells expressing NeuN across groups. **c** The bar chart compares net hippocampal neurogenesis in the SGZ-GCL of the hippocampus across groups. **d** Figures illustrate examples of DCX^+^ newly born neurons from naïve control, GWI-VEH, and GWI-CBD groups (scale bar = 25 µm). The bar chart compares the number of DCX^+^ neurons between different groups. ^**^P < 0.01, ^****^P < 0.0001, ns non-significant. Please refer to Table S3 in Additional file 1 for detailed statistical information. CBD cannabidiol, BrdU 5’-bromodeoxyuridine, VEH vehicle, NeuN neuron-specific nuclear antigen, DCX doublecortin, ML molecular layer, GCL granule cell layer, SGZ subgranular zone

Using BrdU labeling, we quantified the numbers of newly born cells and neurons in the 8th week of VEH/CBD treatment and survived for 8 weeks. Figure 8a displays examples of BrdU-labeled newly born cells. Examples of BrdU^+^ newly born cells that differentiated into mature neurons from naïve control, GWI-VEH, and GWI-CBD groups are shown with dual immunofluorescence for BrdU and NeuN (Fig. 8b). One-way ANOVA analysis comparing the total number of BrdU^+^ newly born cells and net neurogenesis in the SGZ-GCL across groups revealed significant differences between the groups (*P* < 0.0001; Fig. 8a, c). However, there were no group differences in the differentiation of newly born cells into neurons between groups (*P* > 0.05, Fig. 8b). Post hoc tests indicated that the GWI-VEH group had decreased numbers of newly born cells and net neurogenesis compared to the naïve control group (*P* < 0.0001; Fig. 8a, c). However, CBD treatment increased these measures of neurogenesis in GWI rats compared to the GWI-VEH group (*P* < 0.01; Fig. 8a, c). Thus, a 9-week CBD intervention could enhance hippocampal neurogenesis in GWI rats.

We also quantified the status of hippocampal neurogenesis after 16 weeks of either VEH or CBD treatment by assessing the number of DCX^+^ newly born neurons in the SGZ-GCL. The morphology and distribution of DCX^+^ neurons in the SGZ-GCL from different groups are depicted in Fig. 8d. One-way ANOVA analysis revealed significant differences in DCX^+^ new neuron counts among the groups (*P* < 0.01, Fig. 8d). The GWI-VEH group exhibited a lower number of DCX^+^ newly born neurons compared to the naïve group (*P* < 0.01, Fig. 8d). However, CBD treatment restored the levels of DCX^+^ new neurons to those observed in the naïve control group (*P* > 0.05, Fig. 8d). Thus, 16 weeks of CBD treatment can enhance hippocampal neurogenesis to levels comparable to those seen in naïve rats.

## Discussion

The findings of this study provide the first evidence demonstrating that Epidiolex^®^, an FDA-approved CBD, effectively alleviates cognitive and mood impairments, hyperalgesia, oxidative stress, neuroinflammatory signaling cascades, and declines in hippocampal neurogenesis in a rat model of chronic GWI without impacting motor function. Given that chronic oxidative stress and neuroinflammation, along with decreased neurogenesis, are likely to be critical factors contributing to persistent cognitive and mood impairments in GWI patients, the ability of CBD to regulate signaling cascades that perpetuate chronic oxidative stress and neuroinflammation holds significant implications for the treatment of veterans suffering from chronic GWI.

### Mechanisms of CBD-mediated modulation of oxidative stress

Our previous studies have analyzed the brain tissues of rats with chronic GWI at various time points. These studies have identified persistent oxidative stress as one of the primary features of the pathophysiology of chronic GWI [16, 28, 30, 31]. Evidence of oxidative stress in chronic GWI includes elevated levels of MDA and PCs, reduced antioxidants, and increased expression of genes encoding proteins linked to heightened oxidative stress, mitochondrial dysfunction, and dysregulated nuclear factor erythroid 2-related factor 2 (Nrf-2) signaling [16, 28]. This study has shown that CBD can mitigate oxidative stress by reducing concentrations of MDA and PCs while increasing levels of the antioxidant enzyme CAT. The effectiveness of CBD treatment in reducing oxidative stress depends on its duration and dose [69]. CBD indirectly reduces oxidative stress by regulating intracellular pathways such as inflammation [70]. It also directly affects cellular redox status by interrupting free radical chain reactions, chelating transition metal ions, weakening lipid peroxidation, and boosting antioxidant enzyme activity [69–71]. The hydroxyl groups, the pentyl chain in the phenolic ring, and the methyl group on the cyclohexene ring of CBD are responsible for its direct redox-modulating effect [42]. Furthermore, CBD regulates Nrf2 transcriptional activity by downregulating inhibitors such as Kelch ECH associating protein 1 (Keap1) and transcriptional repressor BTB and CNC homology 1 (Bach1), while upregulating activators including Krüppel-associated box-associated protein 1 (KAP1), cyclin-dependent kinase inhibitor p21, and sequestosome-1 (p62) [72]. Additionally, CBD can reduce oxidative stress by altering the endocannabinoid concentration [69]. The current study has demonstrated that CBD is capable of reducing neuroinflammation, lipid peroxidation, and protein oxidation, while simultaneously increasing the antioxidant enzyme CAT. Interestingly, CBD treatment did not have an impact on the concentration of anandamide, suggesting that it can effectively alleviate oxidative stress in GWI without interfering with the endogenous endocannabinoid system. This finding differs from the results in a previous study examining the effects of CBD in schizophrenia patients, where doses escalated to 800 mg/d within the first week and continued for 3 weeks [73]. The discrepancy between this prior study and our current research regarding the effects of exogenous CBD treatment on endogenous anandamide likely stems from variations in species and dosages employed.

### Mechanisms of CBD-mediated modulation of chronic neuroinflammation

Previous studies have consistently reported that chronic neuroinflammation is a prominent feature of GWI in the brain [2, 17, 28–31]. In GWI rats, chronic neuroinflammation is characterized by persistent astrocyte hypertrophy and increased percentages of activated microglia presenting CD68 and NLRP3 inflammasome complexes, elevated levels of proteins involved in NLRP3 inflammasome activation, as well as heightened concentrations of proinflammatory cytokines. Indeed, GWI rats receiving VEH treatment in this study demonstrated most of the above pathophysiological changes in the hippocampus. However, the hippocampus of CBD-treated GWI rats displayed normalization of astrocyte hypertrophy, percentages of microglia with CD68 or NLRP3 inflammasome complexes, levels of proteins mediating NLRP3 inflammasome activation (NF-κB p65, NLRP3, ASC, and cleaved caspase-1), concentrations of end products of activated NLRP3 inflammasomes (IL-1β and IL-18), as well as levels of proteins activating JAK/STAT signaling (IL-6, IFN-γ, p-JAK-1, and p-STAT1), which were restored to naïve control levels. These findings indicate that CBD can effectively attenuate neuroinflammatory signaling in chronic GWI.

CBD treatment can reduce neuroinflammation through multiple mechanisms [74–79]. Firstly, it exerts an inhibitory effect on NF-κB signaling, which is responsible for activating genes encoding proinflammatory cytokines such as IL-1, IL-6, TNF-α, and IFN-γ, as well as NLRP3 inflammasome formation [74–76, 78, 79]. Several studies have demonstrated that CBD can inhibit the NF-κB p65 element involved in this process. Consistent with these findings, long-term CBD treatment to GWI rats in this study normalized the levels of NF-κB p65, IL-1β, IL-6, and IFN-γ to those observed in naïve control levels. Secondly, CBD directly inhibits NLRP3 inflammasome activation [74, 75, 79], which plays a significant role in chronic neuroinflammation associated with GWI [30, 31]. This effect is mediated through the inhibition of genes encoding NLRP3 and caspase-1 (mediators of NLRP3 inflammasome activation), as well as IL-18 and IL-1β (end products of NLRP3 inflammasome activation), Toll-like receptor adaptor myeloid differentiation primary response 88 (MYD88), IFN-γ receptors 1 and 2 (IFNGR1 and IFNGR2), and mitogen-activated protein kinases [74, 75, 79]. Notably, CBD exhibits comparable efficacy to MCC950, a small molecule inhibitor targeting NLRP3, in terms of inhibiting NLRP3 inflammasome activation and production of IL-1β [79]. The results obtained from this study are consistent with the aforementioned activities attributed to CBD since it reduced both the incidences of microglia containing NLRP3 inflammasome complexes and the concentrations of NF-κB p65, NLRP3, ASC, cleaved caspase-1, IL-1β, and IL-18 within the hippocampus.

Another mechanism by which CBD can reduce neuroinflammation is through modulating JAK/STAT signaling [78]. Previous in vitro studies have shown that CBD can downregulate JAK/STAT pathways by decreasing the concentrations of IL-6 and IFN-γ [78], which are the activators of JAK/STAT signaling [80–82]. Studies conducted on models of arthritis, multiple sclerosis, and type 1 diabetes have also shown the ability of CBD to inhibit the JAK/STAT activator IFN-γ [73, 83–86]. The present study has validated the efficacy of long-term CBD treatment in suppressing the activation of JAK/STAT signaling, as evidenced by reduced levels of IL-6, IFN-γ, p-JAK-1, and p-STAT1. Given that phosphorylation of STAT1 leads to robust proinflammatory effects, diminished phosphorylation of STAT1 observed in this study confirmed the potential of CBD for reducing neuroinflammation through modulation of JAK/STAT signaling. Thus, prolonged administration of CBD treatment holds significant promise for mitigating chronic neuroinflammation in GWI by attenuating activated microglia, NLRP3 inflammasome activity, and JAK/STAT signaling pathways. These findings align with previous research suggesting that CBD possesses anti-neuroinflammatory properties via inhibition of proinflammatory cytokines TNF-α, IL-1β, IL-6, and IFN-γ [87–89].

### Potential mechanisms of CBD-mediated improvements in hippocampal neurogenesis

This study also demonstrated the neurogenic effects of CBD in chronic GWI. These effects were evident through an increase in the number of newly born cells (i.e., dentate granule cells) labeled with BrdU, which were generated during the 7th week of CBD treatment and survived for extended periods in the GCL (net hippocampal neurogenesis). Additionally, there was an increase in DCX^+^ newly born neurons observed in the SGZ-GCL, indicating an improved status of neurogenesis following 16 weeks of CBD treatment. While it is known that decreased oxidative stress and neuroinflammation mediated by CBD can increase neurogenesis by enhancing the proliferation of stem/progenitor cells in the hippocampus because of a more favorable microenvironment [90, 91], it is important to consider the potential direct effects of CBD on improving hippocampal neurogenesis. This possibility arises from the activity of CBD on cannabinoid receptors 1 and 2 (CB1 and CB2), which have been shown to influence adult neurogenesis [92, 93]. Previous studies have also demonstrated the dose-dependent beneficial effects of CBD on the generation, survival, and maturation of newly born neurons in the hippocampus [94–97]. Furthermore, CBD treatment has been found to elevate the brain-derived neurotrophic factor concentration, a critical factor capable of enhancing hippocampal neurogenesis [98–100]. Moreover, CBD can inhibit glycogen synthase kinase-3β, a negative regulator involved in maintaining hippocampal neurogenesis through the Wnt/β-catenin pathway [44, 45]. Therefore, multiple mechanisms may contribute to enhanced neurogenesis observed in GWI rats as a result of CBD administration. However, further studies are required to determine the specific mechanisms underlying CBD-mediated increased neurogenesis in GWI.

### Functional impacts of CBD-mediated reduced oxidative stress and neuroinflammation and increased neurogenesis in the hippocampus

Our findings also provide evidence supporting the efficacy of CBD in improving cognitive and mood functions, as well as reducing hyperalgesia in a rat model of chronic GWI. Notably, improvements were observed in hippocampus-dependent cognitive tasks such as object location memory and pattern separation function [55, 56]. Additionally, CBD treatment resulted in enhanced associative recognition memory, a cognitive task that relies on the integrity of multiple brain regions including the medial prefrontal cortex, hippocampus, and perirhinal cortex [101]. Furthermore, CBD administration reversed anhedonia and mitigated hyperalgesia in GWI rats. The amelioration of cognitive and mood impairments following CBD treatment is likely attributed to its antioxidant properties, anti-inflammatory effects, and promotion of neurogenesis. This hypothesis is supported by previous studies demonstrating consistent cognitive and mood deficits associated with conditions characterized by increased oxidative stress, chronic neuroinflammation, and reduced hippocampal neurogenesis [28, 30, 31, 102, 103]. Moreover, since no significant differences were observed in body weights between the GWI-VEH and GWI-CBD groups during the 16-week treatment period, it is unlikely that the alleviation of anhedonia in the GWI-CBD group compared to the GWI-VEH group can be attributed to calorie-seeking behavior. Instead, this finding suggests that anhedonia relief may be due to reductions in neuroinflammation along with maintenance of higher levels of hippocampal neurogenesis. Persistent oxidative stress can lead to neuroinflammation, while chronic neuroinflammation can contribute to continuous oxidative stress through increased release of reactive oxygen species. While either oxidative stress or neuroinflammation alone can induce cognitive and mood impairments, their combined presence, as seen in chronic GWI, exacerbates their detrimental effects on cognitive and mood function.

The hyperalgesia observed in GWI rats in this study is consistent with several investigations demonstrating that exposure to GWI-related chemicals, such as PB, PER, and chlorpyrifos, can lead to chronic pain in rodent models of GWI [104–108]. Notably, studies have indicated that the presence or absence of PB during the exposure period determined whether clinical signs of chronic pain would endure. Changes in the expression of voltage-dependent sodium channel 1.9 and transient receptor potential vanilloid-1 (TRPV1) and nociceptor excitability are associated with chronic pain related to PB exposure [106]. Numerous clinical and preclinical studies have shown that CBD can alleviate pain through its antihyperalgesic effects, primarily by activating TRPV1 [109–112]. Additionally, a study has demonstrated the ability of CBD to increase the withdrawal threshold to mechanical stimulation in neuropathic rats via TRPV1 receptors [113]. This study provides evidence that long-term CBD treatment in GWI rats can elevate paw withdrawal thresholds, supporting the antihyperalgesic effect of CBD. However, further research is necessary to determine whether increased TRPV1 expression underlies the alleviation of hyperalgesia mediated by CBD in GWI rats.

## Conclusions and future directions

This study has demonstrated that oral administration of an FDA-approved CBD (Epidiolex^®^) for 16 weeks can improve cognitive and mood function, as well as alleviate chronic pain in a rat model of GWI. Moreover, the study revealed that functional improvements mediated by CBD were associated with diminished oxidative stress and inflammatory signaling cascades such as NLRP3 inflammasome and JAK/STAT pathway, along with enhanced neurogenesis in the hippocampus. Our previous studies have shown that compounds with anti-inflammatory and antioxidant properties, including curcumin [17], melatonin [30], and monosodium luminol [28], can also improve brain function and reduce neuroinflammation in rats with chronic GWI. However, these studies required relatively higher doses of these compounds [curcumin at 30 mg/(kg·d), i.p; melatonin at 40–80 mg/(kg·d), oral; and monosodium luminol at 160 mg/(kg·d), oral]. In contrast to this finding, the current study found multiple beneficial effects of CBD at a relatively lower dose of 20 mg/kg [equivalent to a human dose of approximately 3.2 mg/(kg.d)]. Therefore, CBD treatment could be a viable option either alone or in combination with existing treatments after ruling out potential drug-drug interactions.

The limitations of this study include the exclusive testing of CBD’s efficacy in male rats. Future studies on CBD for chronic GWI should investigate whether similar effects can be observed in females as well. Additionally, it is crucial to elucidate the mechanisms through which CBD suppresses neuroinflammation, enhances neurogenesis, and improves cognitive and mood function in rats with chronic GWI. Another aspect to consider is the potential adverse effect of suicidal ideation associated with CBD therapy. However, it is important to note that FDA warnings regarding precautions for suicidality published alongside the Epidiolex manual were based on a presumed adverse effect rather than actual data demonstrating suicidal ideation in patients taking CBD [114]. Furthermore, these warnings primarily focused on the potential adverse interaction between CBD and anti-epileptic drugs [114]. To date, no study has reported an increase in suicidality among any clinical population solely using CBD [115]. Nonetheless, future clinical trials investigating the use of CBD in veterans with GWI should be carefully monitored for potential suicidal ideation. Overall, findings from this animal model study suggest that long-term oral administration of CBD holds promise to alleviate cognitive and mood impairments as well as chronic pain experienced by veterans suffering from GWI.

### Supplementary Information

**Additional file 1:** Results. **Fig. S1** Fifteen-minute of restraint stress daily for 28 d in naïve rats does not impair object recognition memory. **Fig. S2** Fifteen-minute of restraint stress daily for 28 d in naïve rats does not impair their pattern separation ability. **Fig. S3** Fifteen-minute of restraint stress daily for 28 d in naïve rats does not cause anhedonia. **Fig. S4** Fifteen-minute of restraint stress daily for 28 d in naïve rats does not cause microglia activation. **Fig. S5** Fifteen-minute of restraint stress daily for 28 d in naïve rats does not cause changes in hippocampal neurogenesis. **Fig. S6** Comparison of weekly body weights for over 16 weeks in GWI rats that received vehicle (GWI-VEH) or CBD (GWI-CBD) during the treatment regimen. **Table S1** List of ELISA kits and reagents employed in the study. **Table S2** List of primary and secondary antibodies employed in the study. **Table S3** Statistical data from one-way ANOVA analysis presented in bar charts of different figures. **Table S4** Statistical data from unpaired, two-tailed, t-tests presented in bar charts of different figures. **Table S5** Statistical data from two-way repeated measures ANOVA

## Data Availability

Source data are included in this original research article. Any additional data requests are available from the corresponding author upon request.

## Abbreviations

AF: Area fraction
ASC: Apoptosis-associated speck-like protein containing a C-terminal caspase recruitment domain
ANOVA: Analysis of variance
Bach1: BTB and CNC homology 1
BrdU: 5’-Bromodeoxyuridine
CAT: Catalase
CBD: Cannabidiol
CD68: Cluster of differentiation 68
DCX: Doublecortin
DEET: N,N-Diethyl-meta-toluamide
DI: Discrimination index
DG: Dentate gyrus
FDA: Food and Drug Administration
FO: Familiar object
FO on P2: Familiar object on pattern 2
GFAP: Glial fibrillary acidic protein
GWI: Gulf War Illness
IBA-1: Ionized calcium-binding adapter molecule-1
IFN-γ: Interferon-γ
IL: Interleukin
IFNGR: Interferon-gamma receptor
JAK/STAT: Janus kinase/signal transducers and activators of the transcription
Keap1: Kelch ECH associating protein 1
KAP1: Kruppel-associated box-associated protein 1
MDA: Malondialdehyde
MYD88: Myeloid differentiation primary response 88
NeuN: Neuron specific nuclear antigen
NF-κB: Nuclear factor-kappa B
NLRP3: NOD-, LRR- and pyrin domain-containing protein 3
NO: Novel object
NO on P2: Novel object on pattern 2
Nrf2: Nuclear factor erythroid 2 related factor 2
OFT: Open field test
OIFP: Objects in familiar place
OINP: Objects in novel place
OIPT: Object-in-place test
OLT: Object location test
PB: Pyridostigmine bromide
PCs: Protein carbonyls
PER: Permethrin
p-JAK-1: Phosphorylated janus kinase-1
PST: Pattern separation test
SOD: Superoxide dismutase
SPR: Sucrose preference rate
SPT: Sucrose preference test
SGZ-GCL: Subgranular zone-dentate granule cell layer
p-STAT-1: Phosphorylated signal transducer and activator of transcription-1
TNF-α: Tumor necrosis factor-α
TOETs: Total object exploration times
TRPV1: Transient receptor potential vanilloid 1
VEH: Vehicle
