# Supplementary material for: FDA-approved cannabidiol [Epidiolex®] alleviates Gulf War Illness-linked cognitive and mood dysfunction, hyperalgesia, neuroinflammatory signaling, and declined neurogenesis
Source: Mil Med Res. 2024 Aug 22;11:61. doi: 10.1186/s40779-024-00563-2 (PMC11340098; doi:10.1186/s40779-024-00563-2)
Supplement: Supplementary file 1 — Additional file 1: Results. Fig. S1 Fifteen-minute of restraint stress daily for 28 d in naïve rats does not impair object recognition memory. Fig. S2 Fifteen-minute of restraint stress daily for 28 d in naïve rats does not impair their pattern separation ability. Fig. S3 Fifteen-minute of restraint stress daily for 28 d in naïve rats does not cause anhedonia. Fig. S4 Fifteen-minute of restraint stress daily for 28 d in naïve rats does not cause microglia activation. Fig. S5 Fifteen-minute of restraint stress daily for 28 d in naïve rats does not cause changes in hippocampal neurogenesis. Fig. S6 Comparison of weekly body weights for over 16 weeks in GWI rats that received vehicle (GWI-VEH) or CBD (GWI-CBD) during the treatment regimen. Table S1 List of ELISA kits and reagents employed in the study. Table S2 List of primary and secondary antibodies employed in the study. Table S3 Statistical data from one-way ANOVA analysis presented in bar charts of different figures. Table S4 Statistical data from unpaired, two-tailed, t-tests presented in bar charts of different figures. Table S5 Statistical data from two-way repeated measures ANOVA [file 40779_2024_563_MOESM1_ESM.pdf]

Results

Adult rats subjected to stress alone (i.e., 15 min of restraint stress daily for 28 d) did not develop cognitive and mood impairments, activated microglia, or a decline in neurogenesis in the hippocampus

Animals and study design

A cohort of 8-week-old male Sprague Dawley rats (n = 10) received restraint stress for 15 min once daily over 28 d. Another cohort of age-matched animals housed in the vivarium served as naïve controls (n = 10). Sixteen weeks after the restraint stress regimen, animals in both groups were interrogated with a series of neurobehavioral tests. After completion of neurocognitive tests, these rats were deeply anesthetized with isoflurane, and their brains were fixed with 4% paraformaldehyde via intracardiac perfusions. Fixed brain tissues were processed for immunohistochemical and immunofluorescence studies. The Institutional Animal Care and Use Committee of Texas A&M University approved all studies conducted in this investigation (Animal Use Protocol number: 2022-0166D).

Fifteen-minute of restraint stress daily for 28 d in naïve rats does not lead to object recognition memory impairment

To evaluate a recognition memory task that depends on the function of the hippocampus and the perirhinal cortex, each animal underwent a novel object recognition test (NORT), as detailed in our previous studies [1, 2] (**Additional file 1: Fig. S1a**). Animals proficient in object recognition memory tend to explore a novel object (NO) over a familiar object (FO). In trial 3 (performed 30 min after trial 2), naïve control rats preferred the NO vs. the FO ($P < 0.0001$, **Additional file 1: Fig. S1b**). Remarkably, naïve control rats subjected to 15-minute restraint stress daily for 28 d also maintained proficiency for object recognition memory by exploring the NO for prolonged periods than the FO ($P < 0.05$, **Additional file 1: Fig. S1b**). Analysis of the discrimination index (DI) for the NO revealed no differences in object recognition memory

between the age-matched naïve group and the group subjected to the stress ($P > 0.05$, **Additional file 1: Fig. S1c**). Thus, 15 min of restraint stress daily for 28 d in naïve rats does not impair object recognition memory.

Fifteen-minute of restraint stress daily for 28 d in naïve rats does not lead to pattern separation impairment

We next evaluated the pattern separation function, which depends upon the integrity of the dentate gyrus in the hippocampus, including the extent of neurogenesis [3, 4]. The test involved 4 trials with an inter-trial interval of 30 min. The test was performed as described in our previous studies [2]. In pattern separation test (PST), animals proficient in pattern separation spend more time exploring the NO on pattern 2 (NO on P2) than the FO on pattern 2 (FO on P2) in T4 (**Additional file 1: Fig. S2a**). The ability for pattern separation was observed in both naïve rats and naïve rats that underwent 15 min of restraint stress daily for 28 d, as they displayed a higher affinity to explore the NO on P2 vs. the FO on P2 ($P < 0.05$, **Additional file 1: Fig. S2b**). Analysis of the NO on P2 – DI confirmed that pattern separation function did not differ between naïve rats and naïve rats that underwent stress ($P > 0.05$, **Additional file 1: Fig. S2c**). Thus, 15 min of restraint stress daily for 28 d in naïve rats does not impair pattern separation function.

Fifteen-minute of restraint stress daily for 28 d in naïve rats does not lead to anhedonia

The anhedonia was assessed through a sucrose preference test (SPT), as described in our previous reports [2, 5, 6]. In SPT, naïve rats and naïve rats subjected to stress (15 min of restraint stress daily for 28 d) preferred sucrose water over standard water, implying no anhedonia ($P < 0.01$, **Additional file 1: Fig. S3a**). Furthermore, the sucrose preference rate (SPR) of naïve rats and naïve rats subjected to stress were comparable ($P > 0.05$, **Additional file 1: Fig. S3b**). Also, the total fluid consumption did not differ between the two groups ($P > 0.05$, **Additional file 1: Fig. S3b**). Thus, 15 min of restraint stress daily for 28 d in naïve rats does not result in anhedonia.

Fifteen-minute of restraint stress daily for 28 d in naïve rats does not activate microglia in the hippocampus

We analyzed the possible chronic activation of microglia in the hippocampus of naïve rats subjected to stress via visualization of IBA-1^{+} microglia expressing CD68 (a marker of activated microglia) through dual immunofluorescence for IBA-1 and CD68 (**Additional file 1: Fig. S4**). Analysis of the percentage of IBA-1^{+} cells expressing CD68 revealed similar percentages of microglia displaying CD68 between naïve rats and naïve rats that underwent stress ($P > 0.05$, **Additional file 1: Fig. S4**). Thus, 15 min of restraint stress daily for 28 d in naïve rats does not lead to chronic activation of microglia in the hippocampus.

Fifteen-minute of restraint stress daily for 28 d in naïve rats does not alter neurogenesis in the hippocampus

Next, we quantified the status of hippocampal neurogenesis via stereological quantification of doublecortin-positive (DCX^{+}) newly born neurons in the subgranular zone – granule cell layer (SGZ-GCL). The morphology and distribution of DCX^{+} neurons in the SGZ-GCL from both groups are illustrated (**Additional file 1: Fig. S5**). The number of DCX^{+} newly born neurons in the SGZ-GCL did not differ between the naïve group and the naïve group subjected to stress ($P > 0.05$, **Additional file 1: Fig. S5**). Thus, 15 min of restraint stress daily for 28 d in naïve rats does not lead to decreased neurogenesis in the hippocampus.

Cannabidiol (CBD) treatment did not cause weight loss in rats with chronic GWI

Since one of the adverse effects of CBD is decreased appetite, we monitored weekly body weights over 16 weeks in GWI rats that received VEH or CBD during the treatment regimen using repeated measures of two-way ANOVA. Interestingly, we did not observe statistically significant differences in body weights between the two groups during the 16-week treatment period (**Additional file 1: Fig. S6**).

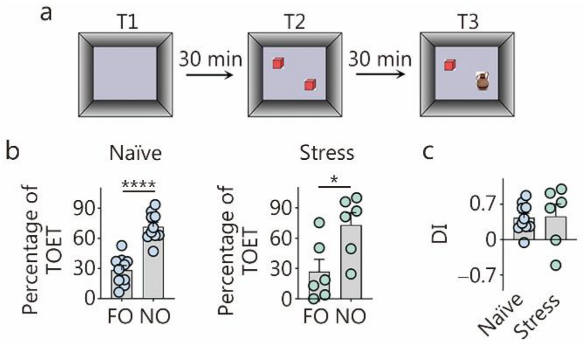

Fig. S1 Fifteen-minute of restraint stress daily for 28 d in naïve rats does not impair object recognition memory. **a** The sequence of trials in a NORT. **b** Percentages of the TOETs spent with the FO and the NO in naïve rats and naïve rats subjected to stress (n = 10). **c** The NO DI between the two groups. $^{*}P < 0.05$, $^{****}P < 0.0001$. Please refer to Table S4 in Additional file 1 for detailed statistical information. NORT novel object recognition test, T trial, FO familiar object, NO novel object, TOET total object exploration times, DI discrimination index

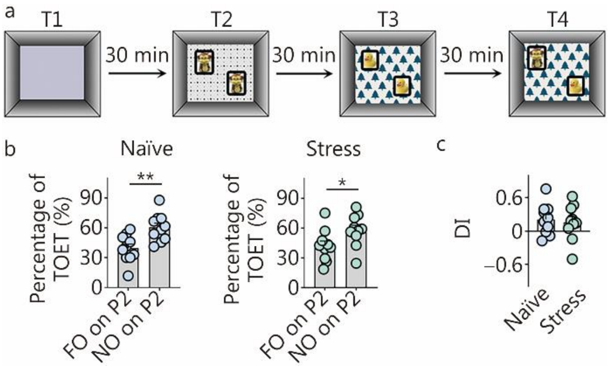

Fig. S2 Fifteen-minute of restraint stress daily for 28 d in naïve rats does not impair their pattern separation ability. **a** The sequence of trials in a PST. **b** Percentages of the TOETs spent with the FO on P2 and the NO on P2 in naïve rats and naïve rats subjected to stress (n= 10). **c** The NO on the P2 DI between the two groups. $^{*}P < 0.05$, $^{**}P < 0.01$. Please refer to Table S4 in Additional file 1 for detailed statistical information. PST pattern separation test, T trial, FO on P2 familiar object on pattern 2, NO on P2 novel object on pattern 2, TOET total object exploration time, DI discrimination index

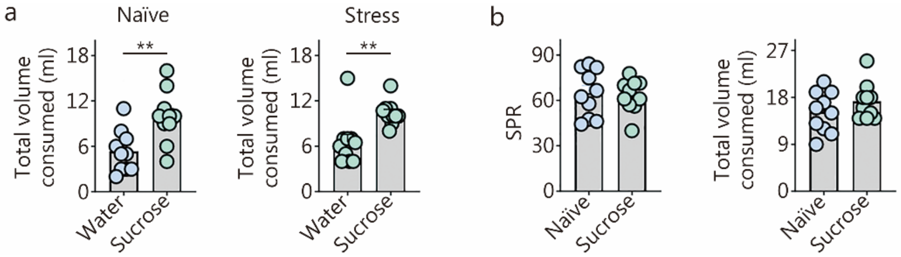

Fig. S3 Fifteen-minute of restraint stress daily for 28 d in naïve rats does not cause anhedonia. **a** The volumes of standard water and sucrose-containing water consumed by animals in naïve rats and naïve rats subjected to stress (n = 10) in the SPT. **b** SPR and total volume consumed between the two groups. $^{**}P <$ 0.01. Please refer to Table S4 in Additional file 1 for detailed statistical information. SPR sucrose preference rate, SPT sucrose preference test

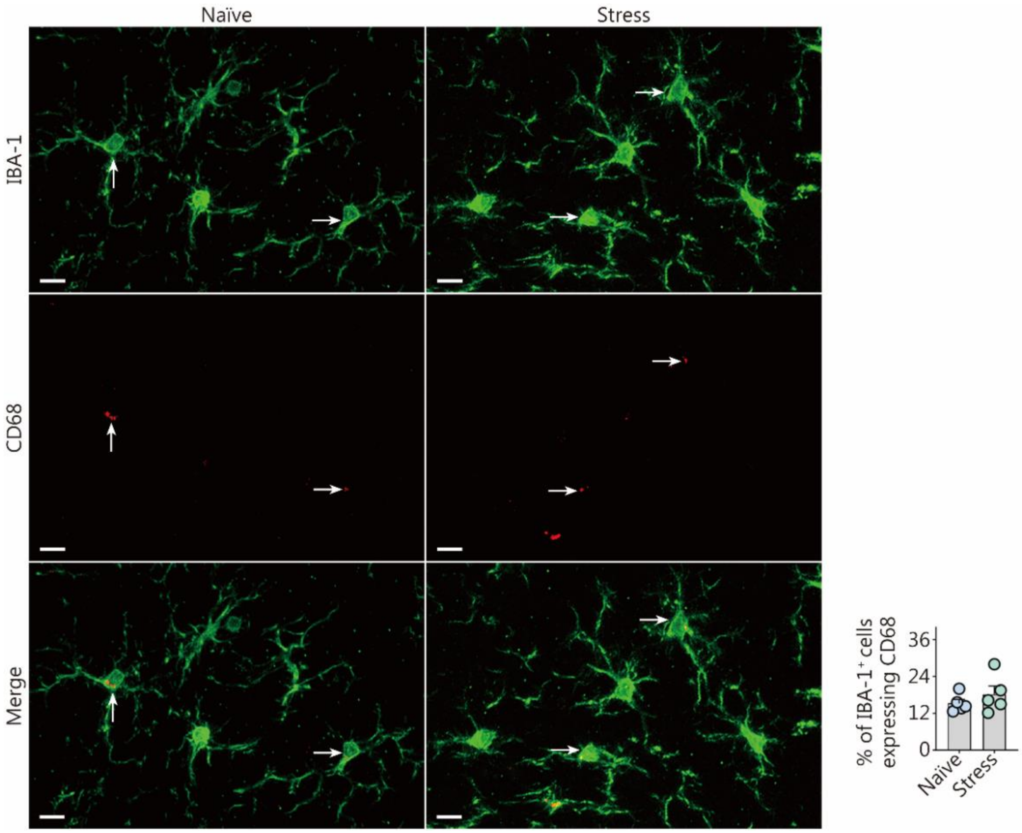

Fig. S4 Fifteen-minute of restraint stress daily for 28 d in naïve rats does not cause microglia activation. The examples of IBA-1$^+$ microglia (green) expressing CD68 (red) in the hippocampal dentate gyrus from a naïve rat and a naïve rat that underwent stress (scale bar = 10 μm). Arrows denote IBA-1$^+$ microglia expressing CD68. The bar chart compares percentages of IBA-1$^+$ microglia expressing CD68 in the hippocampus between the two groups. Please refer to Table S4 in Additional file 1 for detailed statistical information. IBA-1 ionized calcium-binding adapter molecule-1, CD68 cluster of differentiation 68

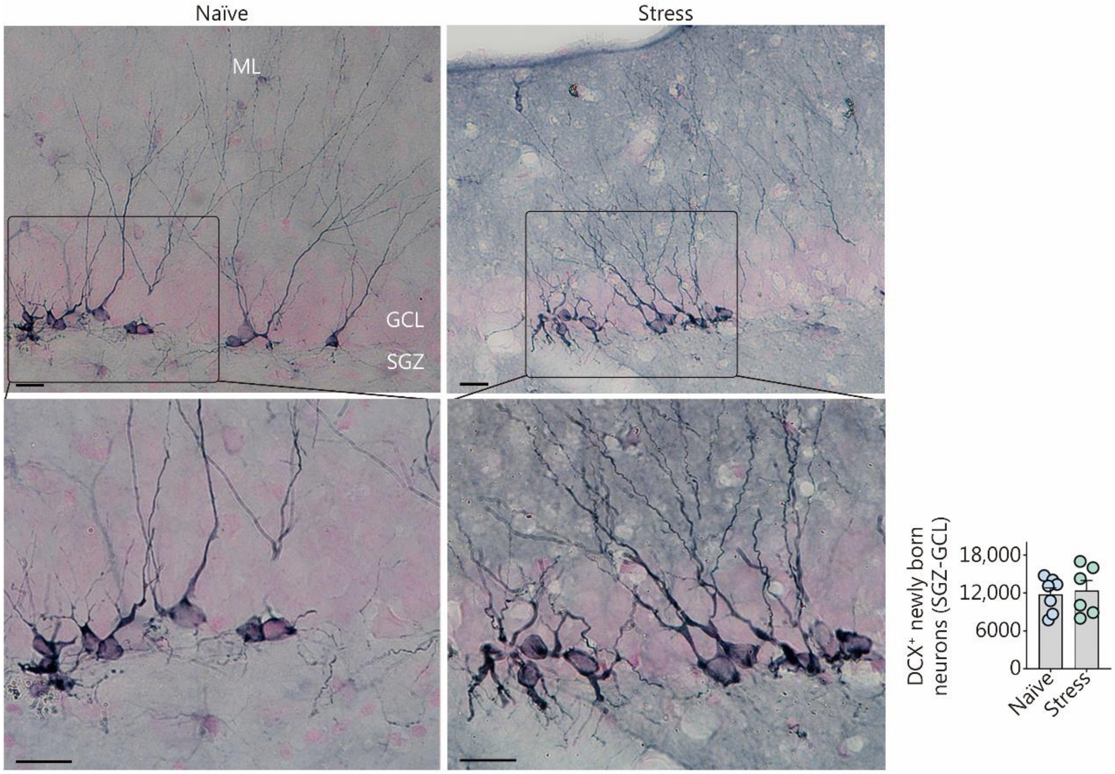

Fig. S5 Fifteen-minute of restraint stress daily for 28 d in naïve rats does not cause changes in hippocampal neurogenesis. Figures illustrate examples of DCX^+ newly born neurons from a naïve rat and a naïve rat subjected to stress (scale bar = 25 μm). The bar chart compares the number of DCX^+ newborn neurons between naïve rats and the naïve rats subjected to stress. Please refer to Table S4 in Additional file 1 for detailed statistical information. ML molecular level, GCL granule cell layer, SGZ subgranular zone, DCX doublecortin

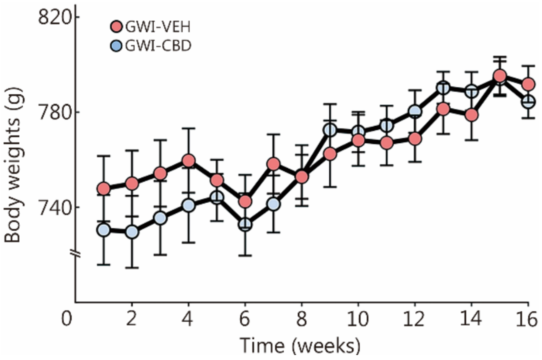

Fig. S6 Comparison of weekly body weights for over 16 weeks in GWI rats that received vehicle (GWI-VEH) or CBD (GWI-CBD) during the treatment regimen. Please refer to Table S5 in Additional file 1 for detailed statistical information. CBD cannabidiol, GWI Gulf War Illness, VEH vehicle

Table S1 List of reagents and ELISA kits employed in the study

ELISA kits	Source	Catalog number
Anandamide	My BioSource San Diego, CA, USA	MBS7254942
ASC	My BioSource, San Diego, CA, USA	MBS7215885
CAT	Cayman Chemicals, Ann Arbor, Michigan, USA	707002
Cleaved caspase-1	Abcam, Cambridge, MA, USA	AB39412
DEET	Chem Service Inc., West Chester, PA, USA	S-12618A1-1ML
IFN-γ	Raybiotech, Peachtree Corners, GA, USA	ELR-IFNg
IL-1β	R&D Systems, Minneapolis, MN, USA	DY501-05
IL-6	R&D Systems Minneapolis, MN, USA	DY406-05
IL-18	R&D Systems, Minneapolis, MN, USA	DY521-05
MDA	Cayman Chemicals, Ann Arbor, Michigan, USA	10009055
NF-κB p65	Aviva Systems Biology, San Diego, CA, USA	OKWB00365
NLRP3	Abcam, Cambridge, MA, USA	AB277086
PCs	Cayman Chemicals, Ann Arbor, Michigan, USA	10005020
Permethrin	Chem Service Inc., West Chester, PA, USA	N-12848-250MG
Pierce BCA reagent kit	Thermo Fisher Scientific, Waltham, USA	23225
p-JAK-1	Raybiotech, Peachtree Corners, GA, USA	PEL-JAK1-Y1022-1
p-STAT-1	Cell Signaling, Danvers, MA, USA	73582
Protease and phosphatase inhibitor	Thermo Fisher Scientific, Waltham, MA, USA	PI78441
Pyridostigmine bromide	Sigma, St. Louis, MO, USA	P9797
SOD	Cayman Chemicals, Ann Arbor, Michigan, USA	706002
Tissue extraction reagent	Thermo Fisher, Waltham, MA, USA	FNN0071

ASC apoptosis associated speck-like protein containing a C-terminal caspase recruitment domain, *CAT* catalase, *DEET* N,N-diethyl-meta-toluamide, *IFN-γ* interferon-γ, *IL* interleukin, *MDA* malondialdehyde, *NF-κB* p65 nuclear factor kappa B p65 subunit, *NLRP3* NOD-, LRR- and pyrin domain-containing protein 3, *PCs* protein carbonyls, *p-JAK-1* phosphorylated Janus kinase-1, *p-STAT-1* phosphorylated signal transducer and activator of transcription-1, *SOD* superoxide dismutase

Table S2 List of primary and secondary antibodies employed in the study

Antibodies	Source	Catalog number
**Primary antibodies**		
Goat anti-IBA-1	Abcam, Cambridge, MA, USA	ab5076
Goat anti-NLRP3	Abcam, Cambridge, MA, USA	AB4207
Mouse anti-BrdU	BD Biosciences, Franklin Lakes, NJ, USA	347580
Mouse anti-CD68	Bio-Rad, Hercules, CA, USA	MCA341R
Mouse anti-ASC	Santa Cruz, Dallas, TX, USA	sc-514414
Rabbit anti-GFAP	Agilent Tech, Santaclara, CA, USA	GA52461-2
Rabbit-anti DCX	Synaptic Systems, Göttingen, Germany	326003
Rabbit anti-IBA-1	Abcam, Cambridge, MA, USA	AB178846
Rabbit anti-NeuN	Millipore, St. Louis, MO, USA	ABN78
**Secondary antibodies**		
ABC Kit	Vector Labs, Burlingame, CA, USA	PK-6100
Goat anti-Rabbit IgG (H + L), Biotinylated	Vector Labs, Burlingame, CA, USA	BA-1000
Donkey anti-goat IgG with Alexa Fluor 488	Invitrogen, Waltham, MA, USA	A11055
Donkey anti-mouse IgG with Alexa Flour 594	Invitrogen, Waltham, MA, USA	A21203
Donkey anti-rabbit IgG with Alexa Fluor 405	Abcam, Cambridge, MA, USA	AB175651
Donkey anti-mouse IgG with Alexa Flour 488	Invitrogen, Waltham, MA, USA	A21202
Donkey anti-rabbit with Alexa Flour 488	Invitrogen, Waltham, MA, USA	A21206
Horse Anti-Goat IgG Antibody (H + L), Biotinylated	Vector Labs, Burlingame, CA, USA	BA-9500
Horse Anti-Mouse IgG Antibody, rat adsorbed (H + L), Biotinylated	Vector Labs, Burlingame, CA, USA	BA-2001

ASC apoptosis-associated speck-like protein containing a C-terminal caspase recruitment domain, *BrdU* 5'-bromodeoxyuridine, *CD68* cluster of differentiation 68, *DCX* doublecortin, *GFAP* glial fibrillary acidic protein, *IBA-1* ionized calcium-binding adapter molecule 1, *NeuN* neuron specific nuclear antigen, *NLRP3* NOD-, LRR- and pyrin domain-containing protein 3

Table S3 Statistical data from one-way ANOVA analysis presented in bar charts of different figures

**Experiment**	**Groups**	**Mean**	**SEM**	**Type of ANOVA**	**Upper 95%CI**	***F*(DFn, DFd)/*H***	**ANOVA *P*-Value**	**Post-hoc *P*-value**
**Fig. 1b,** Distance Traveled (cm)	Naïve<br>GWI-VEH<br>GWI-CBD	1893<br>1672<br>1721	167.4<br>187.8<br>91.1	One-way ANOVA with Tukey's post hoc tests	2247<br>2070<br>1914	F (2, 49) = 2.400	$P > 0.05$	Naïve vs. GWI-VEH: $P > 0.05$<br>GWI-VEH vs. GWI-CBD: $P > 0.05$<br>Naïve vs. GWI-CBD: $P > 0.05$
**Fig. 1b,** Velocity (cm/s)	Naïve<br>GWI-VEH<br>GWI-CBD	6.312<br>5.574<br>5.737	0.5581<br>0.6261<br>0.3037	One-way ANOVA with Tukey's post hoc tests	7.489<br>6.901<br>6.381	F (2, 49) = 0.5715	$P > 0.05$	Naïve vs. GWI-VEH: $P > 0.05$<br>GWI-VEH vs. GWI-CBD: $P > 0.05$<br>Naïve vs. GWI-CBD: $P > 0.05$
**Fig. 2c,** DI	Naïve<br>GWI-VEH<br>GWI-CBD	0.2085<br>-0.1792<br>0.2737	0.1090<br>0.0634<br>0.1302	One-way ANOVA with Tukey's post hoc tests	0.4409<br>-0.0401<br>0.5531	F (2, 41) = 4.777	$P < 0.05$	Naïve vs. GWI-VEH: $P < 0.05$<br>GWI-VEH vs. GWI-CBD: $P < 0.05$<br>Naïve vs. GWI-CBD: $P > 0.05$
**Fig. 2d,** T2-TOET (s)	Naïve<br>GWI-VEH<br>GWI-CBD	42.43<br>49.48<br>29.72	5.299<br>7.011<br>3.909	Kruskal Wallis test with Dunn's post hoc tests	53.79<br>64.76<br>38.10	H = 5.710	$P > 0.05$	Naïve vs. GWI-VEH: $P > 0.05$<br>GWI-VEH vs. GWI-CBD: $P > 0.05$<br>Naïve vs. GWI-CBD: $P > 0.05$
**Fig. 2d,** T3-TOET (s)	Naïve<br>GWI-VEH<br>GWI-CBD	41.81<br>42.36<br>37.11	3.999<br>3.064<br>6.563	One-way ANOVA with Tukey's post hoc tests	50.39<br>49.04<br>51.18	F (2, 40) = 0.3498	$P > 0.05$	Naïve vs. GWI-VEH: $P > 0.05$<br>GWI-VEH vs. GWI-CBD: $P > 0.05$<br>Naïve vs. GWI-CBD: $P > 0.05$
**Fig. 2g,** DI	Naïve<br>GWI-VEH<br>GWI-CBD	0.2781<br>-0.1808<br>0.2639	0.1199<br>0.1014<br>0.1243	One-way ANOVA with Tukey's post hoc tests	0.5352<br>0.0401<br>0.5324	F (2, 39) = 4.815	$P < 0.05$	Naïve vs. GWI-VEH: $P < 0.05$<br>GWI-VEH vs. GWI-CBD: $P < 0.05$<br>Naïve vs. GWI-CBD: $P > 0.05$

Experiment	Groups	Mean	SEM	Type of ANOVA	Upper 95%CI	F(DFn, DFd)/H	ANOVA P-Value	Post-hoc P-value
**Fig. 2h,** T2-TOET (s)	Naïve<br>GWI-VEH<br>GWI-CBD	45.89<br>51.28<br>47.59	3.846<br>8.347<br>5.750	Kruskal Wallis test with Dunn's post hoc tests	54.14<br>69.46<br>60.01	H = 0.01216	$P > 0.05$	Naïve vs. GWI-VEH: $P > 0.05$<br>GWI-VEH vs. GWI-CBD: $P > 0.05$<br>Naïve vs. GWI-CBD: $P > 0.05$
**Fig. 2h,** T3-TOET (s)	Naïve<br>GWI-VEH<br>GWI-CBD	21.45<br>33.51<br>22.62	2.904<br>5.571<br>2.698	Kruskal Wallis test with Dunn's post hoc tests	27.67<br>45.65<br>28.45	H = 4.948	$P > 0.05$	Naïve vs. GWI-VEH: $P > 0.05$<br>GWI-VEH vs. GWI-CBD: $P > 0.05$<br>Naïve vs. GWI-CBD: $P > 0.05$
**Fig. 3c,** DI	Naïve<br>GWI-VEH<br>GWI-CBD	0.2152<br>-0.1917<br>0.1645	0.1586<br>0.1361<br>0.0871	One-way ANOVA with Tukey's post hoc tests	0.5808<br>0.1049<br>0.3501	F (2, 35) = 3.258	P = 0.0504	Naïve vs. GWI-VEH: $P > 0.05$<br>GWI-VEH vs. GWI-CBD: $P > 0.05$<br>Naïve vs. GWI-CBD: $P > 0.05$
**Fig. 3d,** T2-TOET (s)	Naïve<br>GWI-VEH<br>GWI-CBD	36.77<br>43.96<br>39.48	4.139<br>5.735<br>3.615	Kruskal Wallis test with Dunn's post hoc tests	45.79<br>56.58<br>47.19	H = 0.7414	$P > 0.05$	Naïve vs. GWI-VEH: $P > 0.05$<br>GWI-VEH vs. GWI-CBD: $P > 0.05$<br>Naïve vs. GWI-CBD: $P > 0.05$
**Fig. 3d,** T3-TOET (s)	Naïve<br>GWI-VEH<br>GWI-CBD	33.84<br>37.64<br>42.26	5.417<br>4.422<br>4.190	Kruskal Wallis test with Dunn's post hoc tests	46.34<br>47.37<br>51.19	H = 2.726	$P > 0.05$	Naïve vs. GWI-VEH: $P > 0.05$<br>GWI-VEH vs. GWI-CBD: $P > 0.05$<br>Naïve vs. GWI-CBD: $P > 0.05$
**Fig. 3f,** SPR	Naïve<br>GWI-VEH<br>GWI-CBD	74.55<br>50.26<br>64.18	1.998<br>2.588<br>4.158	One-way ANOVA with Tukey's post hoc tests	78.77<br>55.78<br>73.04	F (2, 47) = 16.87	$P <$ 0.0001	Naïve vs. GWI-VEH: $P < 0.0001$<br>GWI-VEH vs. GWI-CBD: $P < 0.01$<br>Naïve vs. GWI-CBD: $P < 0.05$
**Fig. 3g,** Total volume consumed (ml)	Naïve<br>GWI-VEH<br>GWI-CBD	16.39<br>16.50<br>13.44	0.825<br>1.268<br>1.140	Kruskal Wallis test with Dunn's post hoc tests	18.13<br>19.20<br>15.87	H = 5.551	$P > 0.05$	Naïve vs. GWI-VEH: $P > 0.05$<br>GWI-VEH vs. GWI-CBD: $P > 0.05$<br>Naïve vs. GWI-CBD: $P > 0.05$

**Experiment**	**Groups**	**Mean**	**SEM**	**Type of ANOVA**	**Upper 95%CI**	***F*(DFn, DFd)/*H***	**ANOVA *P*-Value**	**Post-hoc *P*-value**
**Fig. 3h,** Paw withdrawal threshold (mN)	Naïve<br>GWI-VEH<br>GWI-CBD	66.30<br>42.71<br>52.46	6.878<br>3.024<br>4.098	One-way ANOVA with Tukey's post hoc tests	81.29<br>49.16<br>61.20	F (2, 42) = 6.171	$P < 0.01$	Naïve vs. GWI-VEH: $P < 0.01$<br>GWI-VEH vs. GWI-CBD: $P > 0.05$<br>Naïve vs. GWI-CBD: $P > 0.05$
**Fig. 4a,** IBA-1$^+$ structures AF (DG)	Naïve<br>GWI-VEH<br>GWI-CBD	24.78<br>26.90<br>24.93	0.9040<br>0.9300<br>0.4864	One-way ANOVA with Tukey's post hoc tests	27.10<br>29.29<br>26.18	F (2, 15) = 2.185	$P > 0.05$	Naïve vs. GWI-VEH: $P > 0.05$<br>GWI-VEH vs. GWI-CBD: $P > 0.05$<br>Naïve vs. GWI-CBD: $P > 0.05$
**Fig. 4a,** IBA-1$^+$ structures AF (CA1)	Naïve<br>GWI-VEH<br>GWI-CBD	21.68<br>24.71<br>20.66	1.0740<br>0.4561<br>0.3507	One-way ANOVA with Tukey's post hoc tests	24.44<br>25.89<br>21.57	F (2, 15) = 8.968	$P < 0.01$	Naïve vs. GWI-VEH: $P < 0.05$<br>GWI-VEH vs. GWI-CBD: $P < 0.01$<br>Naïve vs. GWI-CBD: $P > 0.05$
**Fig. 4a,** IBA-1$^+$ structures AF (CA3)	Naïve<br>GWI-VEH<br>GWI-CBD	21.81<br>25.32<br>23.09	0.9323<br>0.6128<br>0.7174	One-way ANOVA with Tukey's post hoc tests	24.21<br>26.89<br>24.93	F (2, 15) = 5.365	$P < 0.05$	Naïve vs. GWI-VEH: $P < 0.05$<br>GWI-VEH vs. GWI-CBD: $P > 0.05$<br>Naïve vs. GWI-CBD: $P > 0.05$
**Fig. 4a,** IBA-1$^+$ structures AF (EH)	Naïve<br>GWI-VEH<br>GWI-CBD	22.76<br>25.64<br>22.89	0.5299<br>0.3528<br>0.3287	Kruskal Wallis test with Dunn's post hoc tests	24.12<br>26.55<br>23.74	H = 11.42	$P < 0.001$	Naïve vs. GWI-VEH: $P < 0.05$<br>GWI-VEH vs. GWI-CBD: $P < 0.01$<br>Naïve vs. GWI-CBD: $P > 0.05$
**Fig. 4b,** % of IBA-1$^+$ microglia with CD68 (DG)	Naïve<br>GWI-VEH<br>GWI-CBD	19.78<br>23.56<br>21.60	1.764<br>2.076<br>1.792	One-way ANOVA with Tukey's post hoc tests	24.31<br>28.90<br>26.21	F (2, 15) = 1.012	$P > 0.05$	Naïve vs. GWI-VEH: $P > 0.05$<br>GWI-VEH vs. GWI-CBD: $P > 0.05$<br>Naïve vs. GWI-CBD: $P > 0.05$

Experiment	Groups	Mean	SEM	Type of ANOVA	Upper 95%CI	*F*(DFn, DFd)/*H*	ANOVA *P*-Value	Post-hoc *P*-value
**Fig. 4b,** % of IBA-1+ microglia with CD68 (CA1)	Naïve<br>GWI-VEH<br>GWI-CBD	17.68<br>24.71<br>19.33	1.2390<br>0.4561<br>0.9832	One-way ANOVA with Tukey's post hoc tests	20.87<br>25.89<br>21.86	F (2, 15) = 14.98	$P < 0.001$	Naïve vs. GWI-VEH: $P < 0.001$<br>GWI-VEH vs. GWI-CBD: $P < 0.01$<br>Naïve vs. GWI-CBD: $P > 0.05$
**Fig. 4b,** % of IBA-1+ microglia with CD68 (CA3)	Naïve<br>GWI-VEH<br>GWI-CBD	17.65<br>25.48<br>20.25	1.560<br>1.029<br>1.604	Kruskal Wallis test with Dunn's post hoc tests	21.65<br>28.13<br>24.38	H = 8.924	$P < 0.01$	Naïve vs. GWI-VEH: $P < 0.05$<br>GWI-VEH vs. GWI-CBD: $P > 0.05$<br>Naïve vs. GWI-CBD: $P > 0.05$
**Fig. 4b,** % of IBA-1+ microglia with CD68 (EH)	Naïve<br>GWI-VEH<br>GWI-CBD	18.37<br>24.59<br>20.39	1.0050<br>0.4925<br>0.4368	One-way ANOVA with Tukey's post hoc tests	20.95<br>25.85<br>21.52	F (2, 15) = 20.91	$P <$ 0.0001	Naïve vs. GWI-VEH: $P < 0.0001$<br>GWI-VEH vs. GWI-CBD: $P < 0.01$<br>Naïve vs. GWI-CBD: $P > 0.05$
**Fig. 5a,** Number of inflammasomes (per unit area in CA3)	Naïve<br>GWI-VEH<br>GWI-CBD	1.000<br>8.004<br>2.060	0.4472<br>1.4210<br>0.4501	One-way ANOVA with Tukey's post hoc tests	2.242<br>11.950<br>3.310	F (2, 12) = 17.65	$P < 0.001$	Naïve vs. GWI-VEH: $P < 0.001$<br>GWI-VEH vs. GWI-CBD: $P < 0.01$<br>Naïve vs. GWI-CBD: $P > 0.05$
**Fig. 5a,** % of microglia with inflammasomes (CA3)	Naïve<br>GWI-VEH<br>GWI-CBD	4.83<br>42.91<br>15.73	2.177<br>5.842<br>2.927	One-way ANOVA with Tukey's post hoc tests	10.88<br>59.13<br>23.86	F (2, 12) = 24.32	$P <$ 0.0001	Naïve vs. GWI-VEH: $P < 0.0001$<br>GWI-VEH vs. GWI-CBD: $P < 0.01$<br>Naïve vs. GWI-CBD: $P > 0.05$
**Fig. 5b,** NF-κB p65 (OD450)	Naïve<br>GWI-VEH<br>GWI-CBD	0.0989<br>0.1317<br>0.1116	0.005656<br>0.009403<br>0.004609	One-way ANOVA with Tukey's post hoc tests	0.1134<br>0.1559<br>0.1234	F (2, 15) = 5.818	$P < 0.05$	Naïve vs. GWI-VEH: $P < 0.05$<br>GWI-VEH vs. GWI-CBD: $P > 0.05$<br>Naïve vs. GWI-CBD: $P > 0.05$

Experiment	Groups	Mean	SEM	Type of ANOVA	Upper 95%CI	*F*(DFn, DFd)/*H*	ANOVA *P*-Value	Post-hoc *P*-value
Fig. 5b, NLRP3 (ng/mg protein)	Naïve<br>GWI-VEH<br>GWI-CBD	0.3915<br>0.7291<br>0.5690	0.04023<br>0.09806<br>0.03162	Kruskal Wallis test with Dunn's post hoc tests	0.4949<br>0.9812<br>0.6503	$H =$ 10.67	$P < 0.01$	Naïve vs. GWI-VEH: $P < 0.01$<br>GWI-VEH vs. GWI-CBD: $P > 0.05$<br>Naïve vs. GWI-CBD: $P > 0.05$
Fig. 5b, ASC (pg/mg protein)	Naïve<br>GWI-VEH<br>GWI-CBD	17.93<br>43.86<br>20.90	2.796<br>2.666<br>4.899	One-way ANOVA with Tukey's post hoc tests	25.11<br>50.72<br>33.49	F (2, 15) = 15.53	$P < 0.001$	Naïve vs. GWI-VEH: $P < 0.001$<br>GWI-VEH vs. GWI-CBD: $P < 0.01$<br>Naïve vs. GWI-CBD: $P > 0.05$
Fig. 5b, Cleaved caspase-1 (RFU/mg protein)	Naïve<br>GWI-VEH<br>GWI-CBD	14484<br>23361<br>19462	1411<br>3231<br>1513	Kruskal Wallis test with Dunn's post hoc tests	18111<br>31667<br>23352	$H =$ 8.222	$P < 0.01$	Naïve vs. GWI-VEH: $P < 0.05$<br>GWI-VEH vs. GWI-CBD: $P > 0.05$<br>Naïve vs. GWI-CBD: $P > 0.05$
Fig. 5b, IL-1β (pg/mg protein)	Naïve<br>GWI-VEH<br>GWI-CBD	428.3<br>641.1<br>455.9	31.72<br>76.02<br>82.13	Kruskal Wallis test with Dunn's post hoc tests	509.8<br>836.5<br>667.0	$H =$ 7.404	$P < 0.05$	Naïve vs. GWI-VEH: $P > 0.05$<br>GWI-VEH vs. GWI-CBD: $P < 0.05$<br>Naïve vs. GWI-CBD: $P > 0.05$
Fig. 5b, IL-18 (pg/mg protein)	Naïve<br>GWI-VEH<br>GWI-CBD	1496<br>2274<br>1814	85.84<br>185.60<br>93.12	One-way ANOVA with Tukey's post hoc tests	1717<br>2751<br>2053	F (2, 15) = 9.080	$P < 0.01$	Naïve vs. GWI-VEH: $P < 0.01$<br>GWI-VEH vs. GWI-CBD: $P > 0.05$<br>Naïve vs. GWI-CBD: $P > 0.05$
Fig. 5c, MDA (µmol/mg protein)	Naïve<br>GWI-VEH<br>GWI-CBD	3.240<br>12.640<br>7.221	1.050<br>1.566<br>1.566	One-way ANOVA with Tukey's post hoc tests	5.940<br>16.67<br>10.79	F (2, 15) = 12.19	$P < 0.001$	Naïve vs. GWI-VEH: $P < 0.001$<br>GWI-VEH vs. GWI-CBD: $P < 0.05$<br>Naïve vs. GWI-CBD: $P > 0.05$

Experiment	Groups	Mean	SEM	Type of ANOVA	Upper 95%CI	F(DFn, DFd)/H	ANOVA P-Value	Post-hoc P-value
Fig. 5c, PCs (nmol/mg protein)	Naïve<br>GWI-VEH<br>GWI-CBD	16.95<br>26.50<br>15.80	3.175<br>3.134<br>2.043	Kruskal Wallis test with Dunn's post hoc tests	25.11<br>34.55<br>21.05	H = 7.064	$P < 0.05$	Naïve vs. GWI-VEH: $P > 0.05$<br>GWI-VEH vs. GWI-CBD: $P < 0.05$<br>Naïve vs. GWI-CBD: $P > 0.05$
Fig. 5c, SOD (U/mg protein)	Naïve<br>GWI-VEH<br>GWI-CBD	0.9624<br>0.7438<br>0.9665	0.1648<br>0.0688<br>0.1105	Kruskal Wallis test with Dunn's post hoc tests	1.386<br>0.921<br>1.251	H = 3.193	$P > 0.05$	Naïve vs. GWI-VEH: $P > 0.05$<br>GWI-VEH vs. GWI-CBD: $P > 0.05$<br>Naïve vs. GWI-CBD: $P > 0.05$
Fig. 5c, CAT (nmol/min/mg protein	Naïve<br>GWI-VEH<br>GWI-CBD	10.290<br>6.627<br>8.370	0.9703<br>1.0980<br>0.4650	One-way ANOVA with Tukey's post hoc tests	12.780<br>9.451<br>9.565	F (2, 15) = 4.259	$P < 0.05$	Naïve vs. GWI-VEH: $P < 0.05$<br>GWI-VEH vs. GWI-CBD: $P > 0.05$<br>Naïve vs. GWI-CBD: $P > 0.05$
Fig. 6a, IL-6 (pg/mg protein)	Naïve<br>GWI-VEH<br>GWI-CBD	70.13<br>99.45<br>89.26	2.866<br>13.210<br>8.207	Kruskal Wallis test with Dunn's post hoc tests	77.5<br>133.4<br>110.4	H = 7.170	$P < 0.05$	Naïve vs. GWI-VEH: $P < 0.05$<br>GWI-VEH vs. GWI-CBD: $P > 0.05$<br>Naïve vs. GWI-CBD: $P > 0.05$
Fig. 6a, IFN-γ (pg/mg protein)	Naïve<br>GWI-VEH<br>GWI-CBD	4364<br>8244<br>6478	502.5<br>851.0<br>627.7	One-way ANOVA with Tukey's post hoc tests	5656<br>10431<br>8091	F (2, 15) = 8.255	$P < 0.01$	Naïve vs. GWI-VEH: $P < 0.01$<br>GWI-VEH vs. GWI-CBD: $P > 0.05$<br>Naïve vs. GWI-CBD: $P > 0.05$
Fig. 6b, p-JAK-1 (OD450)	Naïve<br>GWI-VEH<br>GWI-CBD	0.1514<br>0.1956<br>0.1675	0.006179<br>0.009900<br>0.005661	One-way ANOVA with Tukey's post hoc tests	0.1673<br>0.2211<br>0.1821	F (2, 15) = 8.935	$P < 0.01$	Naïve vs. GWI-VEH: $P < 0.01$<br>GWI-VEH vs. GWI-CBD: $P < 0.05$<br>Naïve vs. GWI-CBD: $P > 0.05$
Fig. 6b, p-STAT-1 (OD450)	Naïve<br>GWI-VEH<br>GWI-CBD	0.1584<br>0.2324<br>0.1848	0.00792<br>0.02502<br>0.01094	Kruskal Wallis test with Dunn's post hoc tests	0.1788<br>0.2967<br>0.2130	H = 9.836	$P < 0.01$	Naïve vs. GWI-VEH: $P < 0.01$<br>GWI-VEH vs. GWI-CBD: $P > 0.05$<br>Naïve vs. GWI-CBD: $P > 0.05$

Experiment	Groups	Mean	SEM	Type of ANOVA	Upper 95%CI	*F*(DFn, DFd)/*H*	ANOVA *P*-Value	Post-hoc *P*-value
**Fig. 6c,** Anandamide (ng/mg protein)	Naïve<br>GWI-VEH<br>GWI-CBD	4.491<br>4.169<br>5.304	0.2690<br>0.4819<br>0.4627	One-way ANOVA with Tukey's post hoc tests	5.182<br>5.408<br>6.493	F (2, 15) = 1.978	$P > 0.05$	Naïve vs. GWI-VEH: $P > 0.05$<br>GWI-VEH vs. GWI-CBD: $P > 0.05$<br>Naïve vs. GWI-CBD: $P > 0.05$
**Fig. 7,** AF of astrocytic elements (DG)	Naïve<br>GWI-VEH<br>GWI-CBD	18.10<br>21.18<br>20.09	0.7484<br>0.6427<br>0.6132	Kruskal Wallis test with Dunn's post hoc tests	20.02<br>22.96<br>22.67	H = 5.786	$P < 0.05$	Naïve vs. GWI-VEH: $P > 0.05$<br>GWI-VEH vs. GWI-CBD: $P > 0.05$<br>Naïve vs. GWI-CBD: $P > 0.05$
**Fig. 7,** AF of astrocytic elements (CA1)	Naïve<br>GWI-VEH<br>GWI-CBD	17.54<br>21.56<br>19.86	0.9084<br>0.8305<br>0.7179	One-way ANOVA with Tukey's post hoc tests	19.87<br>23.87<br>21.70	F (2, 14) = 5.852	$P < 0.05$	Naïve vs. GWI-VEH: $P < 0.05$<br>GWI-VEH vs. GWI-CBD: $P > 0.05$<br>Naïve vs. GWI-CBD: $P > 0.05$
**Fig. 7,** AF of astrocytic elements (CA3)	Naïve<br>GWI-VEH<br>GWI-CBD	17.85<br>22.23<br>19.95	0.2847<br>0.9257<br>0.8933	One-way ANOVA with Tukey's post hoc tests	18.59<br>24.80<br>22.25	F (2, 14) = 8.422	$P < 0.01$	Naïve vs. GWI-VEH: $P < 0.01$<br>GWI-VEH vs. GWI-CBD: $P > 0.05$<br>Naïve vs. GWI-CBD: $P > 0.05$
**Fig. 7,** AF of astrocytic elements (EH)	Naïve<br>GWI-VEH<br>GWI-CBD	17.83<br>21.50<br>20.27	0.5707<br>0.7558<br>0.5435	Kruskal Wallis test with Dunn's post hoc tests	19.30<br>23.59<br>21.67	H = 10.35	$P < 0.01$	Naïve vs. GWI-VEH: $P < 0.01$<br>GWI-VEH vs. GWI-CBD: $P > 0.05$<br>Naïve vs. GWI-CBD: $P > 0.05$
**Fig. 8a,** $BrdU^+$ cells in SGZ-GCL	Naïve<br>GWI-VEH<br>GWI-CBD	3082<br>1600<br>2098	70.95<br>89.39<br>60.45	One-way ANOVA with Tukey's post hoc tests	3279<br>1848<br>2266	F (2, 12) = 102.3	$P < 0.0001$	Naïve vs. GWI-VEH: $P < 0.0001$<br>GWI-VEH vs. GWI-CBD: $P < 0.01$<br>Naïve vs. GWI-CBD: $P < 0.0001$
**Fig. 8b,** % of $BrdU^+$ cells expressing NeuN	Naïve<br>GWI-VEH<br>GWI-CBD	85.40<br>78.80<br>86.00	2.821<br>4.042<br>2.168	One-way ANOVA with Tukey's post hoc tests	93.23<br>90.02<br>92.02	F (2, 12) = 1.651	$P > 0.05$	Naïve vs. GWI-VEH: $P > 0.05$<br>GWI-VEH vs. GWI-CBD: $P > 0.05$<br>Naïve vs. GWI-CBD: $P > 0.05$

**Experiment**	**Groups**	**Mean**	**SEM**	**Type of ANOVA**	**Upper 95%CI**	***F*(DFn, DFd)/*H***	**ANOVA *P*-Value**	**Post-hoc *P*-value**
**Fig. 8c,** Net neurogenesis	Naïve<br>GWI-VEH<br>GWI-CBD	2633<br>1258<br>1804	112.00<br>91.30<br>64.06	One-way ANOVA with Tukey's post hoc tests	2944<br>1512<br>1981	F (2, 12) = 57.55	$P <$ 0.0001	Naïve vs. GWI-VEH: $P < 0.0001$<br>GWI-VEH vs. GWI-CBD: $P < 0.01$<br>Naïve vs. GWI-CBD: $P < 0.0001$
**Fig. 8d,** DCX^+ neurons in SGZ-GCL	Naïve<br>GWI-VEH<br>GWI-CBD	7543<br>4027<br>5715	852.0<br>457.5<br>270.7	One-way ANOVA with Tukey's post hoc tests	9733<br>5203<br>6411	F (2, 15) = 9.199	$P < 0.01$	Naïve vs. GWI-VEH: $P < 0.01$<br>GWI-VEH vs. GWI-CBD: $P > 0.05$<br>Naïve vs. GWI-CBD: $P > 0.05$

GWI Gulf War Illness, *VEH* vehicle, *CBD* cannabidiol, *BrdU* 5'-bromodeoxyuridine, *NeuN* neuron-specific nuclear antigen, *DCX* doublecortin, *GCL* granule cell layer, *SGZ* subgranular zone, *ASC* apoptosis associated speck-like protein containing a C-terminal caspase recruitment domain, *CAT* catalase, *IFN-γ* interferon-γ, *IL* interleukin, *MDA* malondialdehyde, *NF-κB* p65 nuclear factor kappa B p65 subunit, *NLRP3* NOD-, LRR- and pyrin domain-containing protein 3, *PCs* protein carbonyls, *p-JAK-1* phosphorylated Janus kinase-1, *p-STAT-1* phosphorylated signal transducer and activator of transcription-1, *SOD* superoxide dismutase

Table S4 Statistical data from unpaired, two-tailed, *t*-tests presented in bar charts of different figures

Experiment	Parameters	Mean	SEM	Upper 95%CI	*t*, df/U	*F*, DFn, DFd	*P*-value
Fig. 2b, Naïve, Percentage of TOET	OIFP<br>OINP	38.01<br>62.06	5.350<br>5.356	49.49<br>73.55	$t = 3.177$, df = 28	1.002, 14, 14	$P < 0.01$
Fig. 2b, GWI-VEH, Percentage of TOET	OIFP<br>OINP	56.69<br>43.38	3.670<br>3.680	64.68<br>51.40	$t = 2.559$, df = 24	1.005, 12, 12	$P < 0.05$
Fig. 2b, GWI-CBD, Percentage of TOET	OIFP<br>OINP	36.64<br>63.35	6.513<br>6.518	50.61<br>77.33	$t = 2.899$, df = 28	1.001, 14, 14	$P < 0.01$
Fig. 2f, Naïve, Percentage of TOET	OIFP<br>OINP	37.50<br>62.59	6.695<br>6.685	51.86<br>76.93	$t = 2.653$, df = 28	1.003, 14, 14	$P < 0.05$
Fig. 2f, GWI-VEH, Percentage of TOET	OIFP<br>OINP	56.69<br>43.23	6.031<br>6.022	69.83<br>56.35	$t = 1.580$, df = 24	1.003, 12, 12	$P > 0.05$
Fig. 2f, GWI-CBD Percentage of TOET	OIFP<br>OINP	36.78<br>63.17	6.222<br>6.209	50.22<br>76.58	$t = 3.003$, df = 26	1.004, 13, 13	$P < 0.01$
Fig. 3b, Naïve, Percentage of TOET	FO on P2<br>NO on P2	33.73<br>66.22	5.064<br>5.083	45.41<br>77.95	$t = 4.529$, df = 16	1.008, 8, 8	$P < 0.001$
Fig. 3b, GWI-VEH, Percentage of TOET	FO on P2<br>NO on P2	59.87<br>40.14	7.388<br>7.395	76.13<br>56.42	$t = 1.887$, df = 22	1.002, 11, 11	$P > 0.05$
Fig. 3b, GWI-CBD, Percentage of TOET	FO on P2<br>NO on P2	41.80<br>58.25	4.356<br>4.351	51.08<br>67.53	$t = 2.672$, df = 30	1.002, 15, 15	$P < 0.05$
Fig. 3e, Naïve, Total volume consumed (ml)	Water<br>Sucrose	4.33<br>12.39	0.4354<br>0.7718	5.25<br>14.02	U = 5	-	$P < 0.0001$

Experiment	Parameters	Mean	SEM	Upper 95%CI	*t*, df/U	*F*, DFn, DFd	*P*-value
**Fig. 3e,** GWI-VEH, Total volume consumed (ml)	Water<br>Sucrose	8.000<br>8.500	0.9129<br>0.7130	9.946<br>10.02.	*t* = 0.4317, df = 30	1.639, 15, 15	*P* > 0.05
**Fig. 3e,** GWI-CBD, Total volume consumed (ml)	Water<br>Sucrose	3.588<br>7.647	0.4461<br>1.3140	4.534<br>10.430	*t* = 2.924, df = 32	8.683, 16, 16	*P* < 0.01
**Fig. S1b,** Naïve, Percentage of TOET	FO<br>NO	28.48<br>71.52	4.448<br>4.448	38.54<br>81.58	*t* = 6.843, df = 18	1.000, 9, 9	*P* < 0.0001
**Fig. S1b,** Stress, Percentage of TOET	FO<br>NO	26.93<br>73.07	12.13<br>12.13	58.11<br>104.20	*t* = 2.690, df = 10	1.000, 5, 5	*P* < 0.05
**Fig. S1c,** DI	Naïve<br>Stress	0.4304<br>0.4613	0.08896<br>0.24260	0.6317<br>1.0850	*t* = 0.1422, df = 14	4.460, 5, 9	*P* > 0.05
**Fig. S2b,** Naïve, Percentage of TOET	FO on P2<br>NO on P2	39.51<br>60.49	4.385<br>4.385	49.43<br>70.41	*t* = 3.384, df = 18	1.000, 9, 9	*P* < 0.01
**Fig. S2b,** Stress, Percentage of TOET	FO on P2<br>NO on P2	42.10<br>57.90	5.068<br>5.068	53.57<br>69.36	*t* = 2.204, df = 18	1.000, 9, 9	*P* < 0.05
**Fig. S2c,** DI	Naïve<br>Stress	0.2098<br>0.1580	0.0877<br>0.1014	0.4082<br>0.3873	*t* = 0.3871, df = 18	1.336, 9, 9	*P* > 0.05
**Fig. S3a,** Naïve, Total volume consumed (ml)	Water<br>Sucrose	5.300<br>9.900	0.883<br>1.090	7.296<br>12.370	*t* = 3.280, df = 18	1.525, 9, 9	*P* < 0.01
**Fig. S3a,** Stress, Total volume consumed (ml)	Water<br>Sucrose	6.550<br>10.370	1.023<br>0.493	8.865<br>11.490	U = 10	-	*P* < 0.01

Experiment	Parameters	Mean	SEM	Upper 95%CI	*t*, df/U	*F*, DFn, DFd	*P*-value
**Fig. S3b,** SPR	Naïve<br>Stress	64.87<br>63.29	4.935<br>3.396	76.03<br>70.97	$t = 0.2628$, df = 18	2.112, 9, 9	$P > 0.05$
**Fig. S3b,** Total volume consumed (ml)	Naïve<br>Stress	15.20<br>17.20	1.236<br>1.093	18.00<br>19.67	$t = 1.212$, df = 18	1.279, 9, 9	$P > 0.05$
**Fig. S4,** % of IBA-1^{+} cells expressing CD68	Naïve<br>Stress	15.23<br>18.19	1.287<br>2.724	18.80<br>25.75	$t = 0.9818$, df = 8	4.482, 4, 4	$P > 0.05$
**Fig. S5,** DCX^{+} newly born neurons (SGZ-GCL)	Naïve<br>Stress	11,786<br>12,380	1041<br>1578	14,332<br>1643	$t = 0.3231$, df = 11	1.970, 5, 6	$P > 0.05$

TOET total object exploration times, *DI* discrimination index, *SPR* sucrose preference rate, *IBA-1* ionized calcium-binding adapter molecule-1, *CD68* cluster of differentiation 68, *GCL* granule cell layer, *SGZ* subgranular zone, *DCX* doublecortin, *GWI* Gulf War Illness, *CBD* cannabidiol, *VEH* vehicle

Table S5 Statistical data from two-way repeated measures ANOVA

**ANOVA table**	**Sum of squares (SS)**	**df**	**Mean square (MS)**	***F* (DFn, DFd)**	***P*-value**
Weeks × Treatment	18,997	15	1266	F (15, 435) = 1.023	P = 0.4292
Weeks	181,631	15	12,109	F (3.078, 89.27) = 9.786	P < 0.0001
Treatment	4593	1	4593	F (1, 29) = 0.3152	P = 0.5788

**Difference between column means**	
Mean of GWI-VEH	764.4
Mean of GWI-CBD	758.3
Difference between means	6.115
SE of difference	10.89
95%CI of difference	-16.16 to 28.39

GWI Gulf War Illness, *CBD* cannabidiol, *VEH* vehicle

References

1. Hattiangady B, Mishra V, Kodali M, Shuai B, Rao X, Shetty AK. Object location and object recognition memory impairments, motivation deficits and depression in a model of Gulf War illness. Front Behav Neurosci. 2014;8:78.
2. Madhu LN, Kodali M, Attaluri S, Shuai B, Melissari L, Rao X, et al. Melatonin improves brain function in a model of chronic Gulf War Illness with modulation of oxidative stress, NLRP3 inflammasomes, and BDNF-ERK-CREB pathway in the hippocampus. Redox Biol. 2021;43:101973.
3. Jain S, Yoon SY, Zhu L, Brodbeck J, Dai J, Walker D, et al. Arf4 determines dentate gyrus-mediated pattern separation by regulating dendritic spine development. PLoS One. 2012;7(9):e46340.
4. van Goethem NP, van Hagen BTJ, Prickaerts J. Assessing spatial pattern separation in rodents using the object pattern separation task. Nat Protoc. 2018;13(8):1763-92.
5. Kodali M, Madhu LN, Reger RL, Milutinovic B, Upadhya R, Gonzalez JJ, et al. Intranasally administered human MSC-derived extracellular vesicles inhibit NLRP3-p38/MAPK signaling after TBI and prevent chronic brain dysfunction. Brain Behav Immun. 2023;108:118-34.
6. Ayyubova G, Kodali M, Upadhya R, Madhu LN, Attaluri S, Somayaji Y, et al. Extracellular vesicles from hiPSC-NSCs can prevent peripheral inflammation-induced cognitive dysfunction with inflammasome inhibition and improved neurogenesis in the hippocampus. J Neuroinflammation. 2023;20(1):297.
